# An Autolysis-Derived Trypsin Peptide Disrupts Digestive Homeostasis and Reduces Fitness in *Spodoptera Frugiperda* Caterpillars

**DOI:** 10.1021/acsomega.6c06048

**Published:** 2026-08-07

**Authors:** Daniel Guimarães Silva Paulo, Yaremis Meriño-Cabrera, José Severiche-Castro, Pablo Fernandes Braga, Giovanna dos Santos Pereira, Laryssa Lemos da Silva, Wesley Borges Wurlitzer, Julia Renata Schneider, Eulálio Gutemberg Dias dos Santos, Noeli Juarez Ferla, José Eduardo Serrão, Rafael Júnior de Andrade, Otávio José Bernardes Brustolini, Neilier Rodrigues da Silva Júnior, Humberto Josué Oliveira Ramos, Maria Goreti de Almeida Oliveira

**Affiliations:** † Laboratory of Enzymology and Biochemistry of Proteins and Peptides, Department of Biochemistry and Molecular Biology, 211676 Universidade Federal de Viçosa, UFV, Bioagro/INCT-IPP, Viçosa, Minas Gerais 36570-000, Brazil; ‡ Department of Entomology, 28120 Federal University of Viçosa, Viçosa, Minas Gerais 36570-000, Brazil; § Institute of Biotechnology Applied to Agriculture − BIOAGRO-UFV, Viçosa 36570-000, Brazil; ∥ Departament of Física, Facultad de Ciencias Naturales y Exactas, Universidad del Valle, Cali 760032, Colombia; ⊥ Department of General Biology, Universidade Federal de Viçosa, Viçosa 36570-000, Brazil; # Laboratory of Acarology, Tecnovates, 186081 Universidade do Vale do Taquari, Univates, Lajeado, Rio Grande do Sul 95900-000, Brazil; ∇ National Laboratory for Scientific Computing, Bioinformatics Laboratory − LNCC, Petrópolis, Rio de Janeiro 25651-075, Brazil

## Abstract

The fall armyworm, *Spodoptera frugiperda* J.E. Smith (Lepidoptera: Noctuidae), is a significant agricultural pest, and its management is increasingly impeded by resistance to conventional insecticides, underscoring the need for alternative control strategies. This research examined the effects of the synthetic peptide GORE3 on digestive protease homeostasis and larval performance using an integrated approach that combined molecular dynamics simulations, enzymatic assays, physiological analyses, and histopathology. Comparative analyses employed benzamidine and soybean Kunitz trypsin inhibitor (SKTI) as reference inhibitors. Molecular dynamics simulations demonstrated that the trypsin-GORE3 complex is highly stable, with lower Root Mean Square Deviation (RMSD) values and sustained intermolecular interactions compared with benzamidine, while maintaining a favorable binding free-energy profile. *In vivo* analyses demonstrated that GORE3 induces a dynamic regulation of midgut digestive enzymes, initially suppressing activity, followed by a significant compensatory enhancement in trypsin-like and overall protease activities. Despite this enzymatic compensation, caterpillars exposed to GORE3 has low survival rates, reduced larval weight, prolonged developmental periods, and impaired food conversion efficiency. Nutritional indices indicated a breakdown between digestion and biomass conversion, implying metabolic costs linked to compensatory responses. Histopathological investigations identified midgut damage, including epithelial disruption, epithelial cell disorganization, and structural deterioration with all trypsin inhibitors tested. Oxidative stress responses were detected but played a secondary role relative to digestive dysregulation. Overall, GORE3 disrupts digestive homeostasis by triggering a compensatory yet ineffective proteolytic response, thereby reducing caterpillar fitness. These findings underscore the potential of synthetic peptides targeting digestive proteases as potential bioinsecticides and establish a mechanistic framework linking molecular interactions to organismal effects.

## Graphical abstract

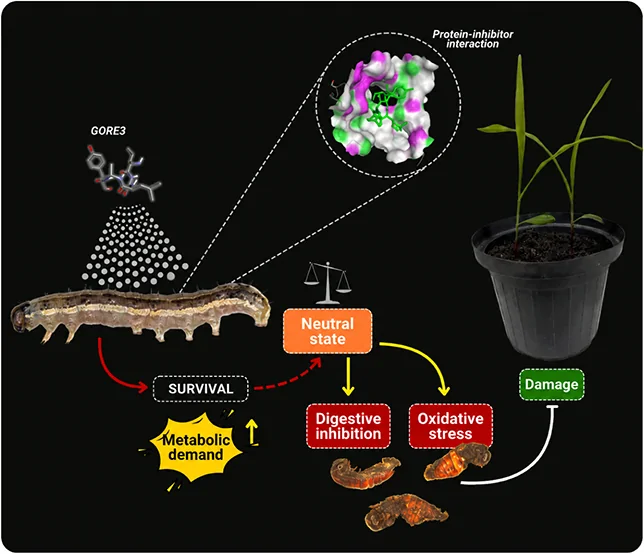

## 1 Introduction

The fall armyworm *Spodoptera frugiperda* (J.E. Smith) (Lepidoptera: Noctuidae) is pests in agriculture worldwide, inflicting significant damage to maize and numerous other economically important crops.^1^ Its notable reproductive capacity, extensive host range, migratory behavior, and remarkable physiological plasticity have facilitated a rapid dispersion and sustained presence in diverse agroecological environments. Although chemical insecticides and Bt-based technologies remain key components to control *S. frugiperda*, the recurrent emergence of resistant populations and environmental concerns associated with intensive pesticide applications necessitate the exploration of alternative strategies that are more selective, sustainable, and compatible with integrated pest management programs.^2–4^

The larval midgut has a vital physiological interface between the insect and its nutritional environment. In lepidopteran caterpillars, digestive proteases, notably trypsin-like serine proteases, are crucial for the hydrolysis of dietary proteins, the acquisition of amino acids, growth, molting, and overall development.^5,6^ Therefore, disruption of digestive proteolysis may impair nutrient absorption and impose metabolic costs, thereby diminishing larval performance.^7–10^ Consequently, trypsin-like enzymes have been extensively studied as biochemical targets for developing protease inhibitors with potential applications in pest management.^11–14^

Protease inhibitors constitute an important category of plant defense molecules and have served as biochemical models for designing compounds that disrupt insect digestion.^12,15–18^ Nonetheless, the effectiveness of these inhibitors is not solely determined by their capacity to inhibit a target enzyme *in vitro*. Insects may initiate compensatory responses, including overproduction of digestive proteases, expression of inhibitor-insensitive isoforms, reorganization of proteolytic activity, and physiological adaptations that partially restore digestion,^12,18,19^ Consequently, a comprehensive understanding of the biological significance of a protease inhibitor necessitates an integrative assessment that correlates molecular binding, enzymatic inhibition, compensatory digestive mechanisms, tissue-level damage, and overall organismal performance.

In this context, comparing inhibitors with distinct biochemical characteristics offers a more robust mechanistic framework than assessing a single molecule in isolation. Benzamidine is a classical small-molecule competitive inhibitor of trypsin-like enzymes and functions as a reference for active-site recognition.^20^ Conversely, soybean Kunitz trypsin inhibitor (SKTI) is a plant-derived protein inhibitor widely used as a model for natural protease inhibition.^21^ GORE3 is a synthetic peptide derived from an autolytic region of trypsin and designed to interact with lepidopteran trypsin-like proteases.^12^ Because autolytic fragments may retain structural features associated with enzyme recognition and regulatory interactions, peptides derived from these regions are promising templates for the development of selective digestive protease inhibitors.^22^ Collectively, these three inhibitors provide a comparative platform to evaluate whether an autolysis-derived synthetic peptide can elicit biological effects comparable to or distinct from those induced by a canonical small-molecule inhibitor and a natural proteinaceous inhibitor.

Previous work showed that GORE3 interacts with *S. frugiperda* trypsin-like proteases through a conserved binding mode, occupying the catalytic cleft and adjacent subsites, with a broader interaction network than benzamidine.^12^ That study also reported that GORE3 acts as a competitive inhibitor and affects larval development, supporting its relevance as a peptide model for studying digestive protease inhibition in lepidopterans. However, docking and enzymatic inhibition alone do not fully explain whether this interaction remains stable under dynamic conditions or whether it is sufficient to trigger persistent digestive dysregulation, oxidative imbalance, intestinal damage, and reduced larval fitness.

Because protein–ligand complexes may explore different conformational substates during molecular dynamics simulations, the use of independent replicas has become an important strategy to improve conformational sampling and reduce the risk of interpreting trajectory-specific fluctuations as representative molecular behavior. Multiple short or independent molecular dynamics simulations have been successfully applied to characterize ligand-induced conformational transitions, binding selectivity, and free-energy trends in biomolecular complexes. For example, Chen et al.^23^ used multiple short molecular dynamics simulations combined with Markov model analysis to investigate ligand-mediated conformational transitions in eukaryotic riboswitches, whereas Chen et al.^24^ applied multiple short molecular dynamics simulations and free-energy predictions to clarify inhibitor selectivity toward BACE1 and BACE2. Therefore, independent replicas provide a more reliable basis for comparing the dynamic stability and energetic behavior of inhibitor-bound complexes.

A critical knowledge gap remains regarding how molecular recognition of trypsin-like enzymes translates into systemic physiological disruption in *S. frugiperda*. Specifically, it is still necessary to determine whether the interaction between GORE3 and trypsin-like proteases leads to stable complex formation during molecular dynamics simulations, favorable binding free-energy profiles, persistent modulation of trypsin-like and total proteolytic activities, oxidative stress responses, impaired nutrient conversion, altered survival, and midgut histopathological damages. Addressing these gaps is particularly important because the biological outcome of protease inhibition depends not only on binding affinity or enzyme inhibition, but also on the insect’s capacity to compensate for digestive stress.^25–28^

The present study employs an integrated approach that combines *in silico*, *in vitro*, and *in vivo* methods to evaluate the effects of GORE3 on digestive protease homeostasis and caterpillar performance in *S. frugipeda*. Comparative analysis was conducted using benzamidine and SKTI as reference inhibitors. It is hypothesized that GORE3, through the formation of stable interactions with *S. frugiperda* trypsin-like proteases, disrupts the homeostasis of digestive proteases and triggers a compensatory proteolytic response that is insufficient to restore normal larval performance. Furthermore, compared to benzamidine and SKTI, GORE3 is expected to induce a distinct physiological signature characterized by changes in digestive enzyme activity, reduced nutritional efficiency, damage to midgut structure, delayed developmental progression, and decreased survival rates. By integrating molecular simulations, enzymatic activity assays, oxidative stress analyses, assessments of nutritional and developmental parameters, and histopathological examinations, this study provides a comprehensive framework for understanding the effects of synthetic protease-inhibitory peptides on the digestive physiology of this important lepidopteran pest.

## 2 Materials and Methods

### 2.1 Molecular Dynamics Simulations, Structural Stability Analysis, and Binding Free-Energy Estimation

Molecular dynamics simulations were performed using GROMACS to evaluate the structural stability, flexibility, interaction persistence, and entropy-uncorrected effective energetic profiles of the complexes formed between *S. frugiperda* trypsin and the inhibitors SKTI, GORE3, and benzamidine. The initial trypsin–inhibitor complexes were selected from the molecular docking study reported by Paulo et al. (2026), in which different *S. frugiperda* trypsin-like isoforms were modeled and evaluated against GORE3 and benzamidine. Because the docking protocol, pose selection criteria, and initial interaction analyses were described in that previous study, the present work focused on the dynamic behavior of representative complexes under explicit-solvent molecular dynamics conditions.

Before molecular dynamics simulations, each trypsin–inhibitor complex was visually inspected to verify the system’s structural integrity, the preservation of the expected binding orientation, and the inhibitor’s location within or near the enzyme’s catalytic cleft. Missing hydrogen atoms were added, protonation states were assigned, and the corresponding topology and coordinate files were generated.

Missing hydrogen atoms were added, and fixed protonation states were assigned before molecular dynamics simulations, following the standard protonation scheme used during topology generation with the AMBER99 force field in GROMACS. Because conventional molecular dynamics simulations do not explicitly impose or dynamically control bulk pH, the protonation states remained fixed throughout the trajectories. Therefore, the simulations should not be interpreted as constant-pH simulations or as a direct representation of the strongly alkaline midgut environment. The protonation scheme was used to provide a consistent, comparative setup for the trypsin–SKTI, trypsin–GORE3, and trypsin–benzamidine complexes, all evaluated under the same computational protocol.

The protein components of the systems, including *S. frugiperda* trypsin, SKTI, and the peptide GORE3, were described using the AMBER99SB-ILDN force field. Because GORE3 is a peptide composed of standard amino acid residues, it was parametrized using the same protein force-field parameters. Benzamidine, which is a nonstandard small-molecule ligand, was parametrized separately. The optimized benzamidine structure was parametrized using the General AMBER Force Field (GAFF). Partial atomic charges were calculated using the semiempirical AM1-BCC method with the Antechamber tool implemented in AmberTools. Subsequently, GROMACS-compatible ligand topologies and parameters were generated using the ACPYPE command-line interface, ensuring consistent assignment of bond, angle, dihedral, and atom-type parameters derived from GAFF.

Each trypsin–inhibitor complex was placed in a cubic box of explicit TIP3P water molecules under periodic boundary conditions. The systems were neutralized and ionized by adding 92 Na^+^ ions and 90 Cl^–^ ions, providing electrostatic stability under the simulated conditions. After solvation and ion addition, each system underwent 1000 steps of energy minimization to remove unfavorable steric contacts and optimize local atomic geometries prior to equilibration.

The minimized systems were gradually equilibrated before production dynamics. First, restrained equilibration was performed under NVT conditions to stabilize the system temperature. Subsequently, NPT equilibration was performed at 310 K and 1 atm, allowing solvent density, ion distribution, and simulation box dimensions to adjust under constant pressure. These equilibration stages ensured progressive relaxation of the solvent, ions, protein, and inhibitors before the unrestrained production simulations.

Production molecular dynamics simulations were carried out for 100 ns for each trypsin–inhibitor complex under NPT conditions. All simulations were performed using a 2 fs integration time step, and covalent bonds involving hydrogen atoms were constrained. Short-range nonbonded interactions were calculated using a 12 Å cutoff, whereas long-range electrostatic interactions were treated using the particle mesh Ewald method. Temperature was controlled using Langevin dynamics, and pressure was maintained using the Langevin piston method. Trajectory coordinates were saved every 10 ps for subsequent structural and energetic analyses.

To improve conformational sampling and reduce the influence of trajectory-specific fluctuations, three independent molecular dynamics replicas were performed for each trypsin–inhibitor complex. The replicas were simulated under identical physical conditions but were initiated with different initial velocity distributions. This strategy allowed each complex to explore conformational space through independent trajectories and provided a more robust basis for comparing the dynamic behavior of the trypsin–SKTI, trypsin–benzamidine, and trypsin–GORE3 complexes. Unless otherwise indicated, the reported profiles correspond to the mean behavior of the three replicas, with the standard deviation used to describe variability among trajectories.

Trajectory analyses were performed to compare the dynamic behavior of the trypsin complexes with SKTI, GORE3, and benzamidine. Before analysis, all frames were aligned to the initial structure using the backbone atoms of the receptor to remove global translational and rotational motions. Structural stability was evaluated by calculating the root-mean-square deviation (RMSD) for the whole complex, the receptor, and the inhibitor. Local flexibility was assessed through root-mean-square fluctuation (RMSF) analysis using the Cα atoms of the receptor. In addition, intermolecular hydrogen bonds between trypsin and each inhibitor were monitored during the trajectories using geometric criteria based on donor–acceptor distance and bond angle. These analyses allowed comparison of the stability, flexibility, compactness, solvent exposure, and persistence of the interaction networks among the three trypsin–inhibitor systems.

Binding free-energy calculations were performed using MM/GBSA and MM/PBSA approaches from representative snapshots extracted from the equilibrated production trajectories. To minimize the influence of the initial structural relaxation stage, only frames from the final 50 ns of each 100 ns simulation, corresponding to the 50–100 ns interval, were considered for energetic analysis. A total of 100 snapshots per replica were selected at regular 0.5 ns intervals. The same snapshot selection protocol was applied to all systems to ensure a consistent comparison among SKTI, GORE3, and benzamidine.

The reported entropy-uncorrected MM/GBSA and MM/PBSA effective interaction-energy estimates correspond to the mean ± standard deviation obtained from the analyzed snapshots. Electrostatic and van der Waals energy components were also extracted and reported as average values. The calculated estimates were interpreted as relative energetic descriptors useful for comparing the consistency of ligand interaction patterns across independent replicas, rather than as absolute predictors of experimental affinity.

For consistency with the MM/GBSA and MM/PBSA end point formalisms, the effective interaction-energy estimate was defined as

$$\Delta G_{Eff}=G_{RL}-G_{R}-G_{L}$$

where *G_RL_*, *G_R_*, and *G*_*L*_correspond to the free energies of the complex, the isolated receptor, and the isolated ligand, respectively.

In the MM/GBSA end point formalisms, the entropy-uncorrected effective interaction-energy estimate can be expressed as

$$\Delta G_{\mathrm{Eff}}=\Delta H-T\Delta S=\Delta E_{\mathrm{MM}}+\Delta G_{\mathrm{sol}}-T\Delta S$$

where Δ*E*_*MM*_represents the gas-phase molecular mechanics energy, Δ*G*_*sol*_ is the solvation free energy, and *T*Δ*S* is the entropic contribution. The molecular mechanics term includes electrostatic (Δ*E*_*ele*_), van der Waals (Δ*E*_*vdW*_), and internal (Δ*E*_*int*_)­energy contributions, whereas the solvation term includes polar solvation, calculated using the generalized Born model, and nonpolar solvation, estimated from the solvent-accessible surface area. In the present study, the entropic term was not included in order to reduce computational cost. Therefore, the reported values are described as entropy-uncorrected MM/GBSA and MM/PBSA effective interaction-energy estimates and were interpreted only as qualitative energetic descriptors of the simulated complexes.

In the present study, the entropic term was not included in order to reduce computational cost, because only relative binding free-energy comparisons were considered. For the MM/GBSA calculations, a solvent dielectric constant of 78.5 and a surface tension constant of 0.03012 kJ mol^–1^ Å^–2^ were used. In addition to the total binding free energy, electrostatic and van der Waals energy components were extracted from the analyzed snapshots and reported as average values for each simulation. Binding free energy values are presented as mean ± standard deviation.

This approach allowed a consistent comparison of the energetic behavior of the trypsin–SKTI, trypsin–benzamidine, and trypsin–GORE3 complexes, while avoiding overinterpretation of the calculated binding free energies as direct experimental affinity constants.

### 2.2 Rearing of the Caterpillars

Eggs of *S. frugiperda* were obtained from an established colony maintained at the Laboratory of Enzymology and Biochemistry of Proteins and Peptides II, located at the Institute of Biotechnology Applied to Agriculture (BIOAGRO), Federal University of Viçosa, Brazil. After egg hatching, caterpillars were individualized and reared on an artificial diet^29^ in trays divided into individual cells. Adult moths were maintained in 200 mm diameter PVC tubes lined with A4 paper sheets and covered with voile fabric. Adults were fed a diet composed of beer, honey, sucrose, ascorbic acid, nipagin, and water.^29^ The solution was supplied onto cotton and placed in a Petri dish (100 × 150 mm). The colony was maintained at 25 ± 1 °C, 60 ± 10% relative humidity, and a 14:10 h (light: dark) photoperiod.

### 2.3 Experimental Design

For the *in vitro* and *in vivo* assays, caterpillars used in the experimental evaluations were obtained from the mass-rearing colony described in Section 2.1. The experimental design comprised four treatments: (a) control (no inhibitor addition); (b) benzamidine (≥97% purity by titration; trypsin and trypsin-like enzyme inhibitor, solid; Sigma-Aldrich, St. Louis, MO, USA); (c) SKTI (protein content 70–90%, determined by the Biuret method; Sigma-Aldrich, St. Louis, MO, USA); and (d) GORE3 (95% purity; Aminotech, Brazil). The identity of the synthetic peptide was confirmed by electrospray ionization mass spectrometry (ESI-MS), as provided in the Supporting Information (Table S1 and Figure S1). A total of 150 individuals were evaluated per treatment, resulting in a total of 600 caterpillars.

For incorporation of protease inhibitors, the artificial diet was subjected to thermal treatment in a water bath for 10 min. Briefly, the PIs addition was performed in the diet at 45 °C, following the protocol described by de Almeida Barros.^30^ Protease inhibitors were incorporated into the artificial diet at a final concentration of 0.1% (w/v), corresponding to the concentration previously reported to produce the highest biological effect.^12^ Third-instar larvae were continuously exposed to the respective treatments throughout the larval period. During the fifth instar, 10 insects per treatment were dissected, and their midguts were collected, transferred to microcentrifuge tubes, and immediately stored at – 80 °C until subsequent enzymatic assays.

### 2.4 *In Vitro* Analyses

#### 2.4.1 Persistence of Trypsin-Like and Total Protease Inhibition by Peptides

To evaluate the persistence of endogenous inhibition of trypsin-like enzymes and total proteolytic activity in the midgut of *S. frugiperda* induced by benzamidine, SKTI, and GORE3, third-instar caterpillars were individualized and exposed to diets containing the respective inhibitors. After 24 h of exposure, 30 caterpillars per treatment were transferred to an inhibitor-free diet and maintained under these conditions until the end of the experiment. Midgut collections were performed at 24, 48, 72, and 96 h, and six caterpillars per treatment were dissected at each time point.

The midguts were meticulously dissected, immediately frozen in liquid nitrogen, and stored at – 80 °C until enzymatic analyses. Each treatment included three biological replicates, with each replicate comprising two pooled midguts. Trypsin-like activity was quantified using the chromogenic substrate L-BApNA. Briefly, 20 μL of enzymatic extract was added to 100 μL of 50 mM Tris-HCl buffer (pH 8.0) containing 20 mM CaCl_2_, followed by the addition of 100 μL of L-BApNA (0.4 mM) prepared in the same buffer. The reaction was conducted, and the release of p-nitroaniline was monitored by measuring the change in absorbance at 410 nm for 2.5 min, with activity expressed as ΔA410/min.^31^

Total proteolytic activity was quantified using azocasein (0.5% w/v) in a 0.1 M Tris-HCl buffer (pH 8.2) supplemented with 20 mM CaCl_2_, maintained at 37 °C. The reaction mixture comprised 80 μL of substrate and 60 μL of enzymatic extract. Following a 30 min incubation, the reaction was halted by adding 240 μL of 10% (w/v) trichloroacetic acid. The samples were vortexed, stored on ice for 15 min, and subsequently centrifuged at 8,000 × g for 5 min at 25 °C to eliminate precipitated material. Afterward, 240 μL of the supernatant was transferred into microtubes containing 280 μL of 1 M NaOH, and the absorbance was then measured at 440 nm.

For both enzymatic activities (trypsin-like and total proteases), three technical replicates were performed for each biological sample.

#### 2.4.2 Oxidative Stress

Total antioxidant capacity was assessed using the ferric reducing antioxidant power (FRAP) assay,^32^ using 2,4,6-tri­(2-pyridyl)-s-triazine (TPTZ) as the chromogenic substrate. The method is based on the reduction of the ferric-TPTZ (Fe^3+^ -TPTZ) complex to the ferrous form (Fe^2+^ -TPTZ). A volume of 10 μL of each midgut extract was added to 190 μL of FRAP reagent, which consisted of 25 mL of acetate buffer (300 mmol L^–1^, pH 3.6), 2.5 mL of TPTZ (10 mmol L^–1^), and 2.5 mL of FeCl_3_·6H_2_O (20 mmol L^–1^). Absorbance was read at 593 nm. The reduction of the Fe^3+^ -TPTZ complex by antioxidants was calculated based on a standard curve of serial dilutions of FeSO_4_·7H_2_O starting at 2 mmol L^–1^. Results were expressed as μmol mg^–1^.

Catalase activity (CAT) was determined as described by Levine et al.^33^ For the reaction, 100 μL of midgut extract was incubated in 2 mL of substrate at pH 7.4 (65 μmol mL-1 hydrogen peroxide in 60 nmol of sodium phosphate buffer, 1.1 g Na_2_HPO_4_, and 0.27 g KH_2_PO_4_ in 100 mL of distilled water) at 30 °C. Absorbance readings were obtained at 240 nm in automatic mode, with one reading per second for 120 s (Molecular Devices SpectraMax ABS Plus). Specific catalase activity values were expressed in μmol s^–1^ mg protein^–1^.

The hydrogen peroxide (H_2_O_2_) content was determined using a spectrophotometric method based on the formation of a peroxomolybdate complex, following reaction with ammonium molybdate.^34^ Samples were prepared in sodium phosphate buffer (50 mM, pH 7.4). For the assay, 5 μL of each sample were added to microplate wells containing 100 μL of phosphate buffer. The reaction was initiated by the addition of hydrogen peroxide and allowed to proceed for 3 min. Subsequently, 150 μL of ammonium molybdate solution (prepared by dissolving 2 g of (NH_4_)_6_Mo_7_O_24_·4H_2_O in 50 mL of phosphate buffer, 50 mM, pH 7.4) was added to stop the reaction and form the peroxomolybdate complex. The absorbance was measured at 374 nm using a microplate reader. A standard curve was prepared using serial dilutions of hydrogen peroxide in phosphate buffer, and the H_2_O_2_ concentrations in the samples were calculated by interpolation from the standard curve. Results were expressed as nmol mL^–1^.

Superoxide dismutase (SOD) activity was determined based on the inhibition of pyrogallol auto-oxidation, monitored spectrophotometrically at 320 nm.^35^ The reaction was performed in a microplate containing 10 μL of midgut extract and 0.1 M phosphate buffer (pH 7.8), and was initiated by adding a pyrogallol solution previously prepared in 0.1 M phosphate buffer (pH 5.4). Absorbance readings were taken at 1 and 3 min, with a 2 min interval between measurements, and the plate was agitated for 10 s before each measurement. Enzyme activity was calculated from the change in absorbance between readings, considering the total reaction volume and the volume of sample used. Results were expressed as U mg^–1^.

Nitric oxide (NO) levels were estimated by measuring nitrite (NO_2_^–^), a stable NO metabolite, using the Griess reaction. Briefly, two stock solutions were prepared: solution A containing 1% (w/v) sulfanilamide in 2.5% (v/v) phosphoric acid (H_3_PO_4_), and solution B containing 0.1% (w/v) N-(1-naphthyl) ethylenediamine dihydrochloride (NED) in 2.5% (v/v) H_3_PO_4_. Immediately before use, the Griess reagent was prepared by mixing solutions A and B in equal volumes. A sodium nitrite (NaNO_2_) standard solution (200 μM) was used to generate a calibration curve by serial dilution in sodium phosphate buffer. For the assay, 50 μL of standards or samples were added to microplate wells, followed by 100 μL of freshly prepared Griess reagent. The mixture was incubated in the dark at room temperature for 10 min, and the absorbance was measured at 570 nm using a microplate reader. Nitrite concentrations were determined by interpolation from the standard curve and expressed relative to the protein content of each sample. Results were expressed as nM mg^–1^.

Protein carbonyl content was determined using the 2,4-dinitrophenylhydrazine (DNPH) method,^36^ which is based on the reaction of carbonyl groups with DNPH. A total of 50 μL of gut extract was added to 0.5 mL of 10 mmol/L DNPH, diluted in 7% HCl. The mixture was vortexed and incubated in the dark at room temperature for 30 min, with occasional shaking. Then, 0.5 mL of cold 10% trichloroacetic acid (TCA) was added, followed by centrifugation at 5000 g for 10 min at 4 °C. The supernatant was discarded, and the pellet was washed three times with 1 mL of a 1:1 (v/v) ethyl acetate: ethanol solution. Finally, 1 mL of 6% SDS was added, and the pellet was solubilized by vortexing. Absorbance was measured at 370 nm, and results were expressed as nmol/mg of protein, based on a molar extinction coefficient of 22 mmol L^–1^ cm^–1^.

The lipid peroxidation was assessed via the thiobarbituric acid-reactive substances (TBARS) assay for malondialdehyde (MDA).^37^ Briefly, 0.2 mL of the gut extracts were homogenized in 0.4 mL of a solution containing trichloroacetic acid (15%), thiobarbituric acid (0.375%), and hydrochloric acid (0.6%). The mixture was incubated in a boiling water bath for 40 min. After cooling on ice, 0.6 mL of butanol was added, followed by vortexing for 2 min and centrifugation at 9000 g for 10 min. The supernatant was used to measure absorbance at 535 nm using a microplate scanning spectrophotometer (Multiskan GO). MDA concentration was determined based on a standard curve of known concentrations of 1,1,3,3-tetramethoxypropane (TMPO), and results were expressed as μmol L^–1^ mg protein^1^.

### 2.5 Survival Analysis

Caterpillar survival analysis was conducted by examining the number of individuals remaining alive over time throughout the entire life cycle of *S. frugiperda*. A total of 130 individuals per treatment were evaluated, resulting in a total of 520 caterpillars, which were monitored daily, and mortality events were recorded. The treatments included 0.1% (w/v) concentrations of GORE3, benzamidine, and SKTI, along with a water control. The number of surviving individuals per treatment was counted daily. Individuals were considered dead when they showed no movement after gentle mechanical stimulation with a fine brush. The survival time of each individual was recorded as the number of days from the beginning of the treatment until death.

### 2.6 Biological Cycle and Body Mass of Larvae and Pupae

The duration of the larval period was determined through daily observations. The period commenced at egg hatching and concluded at pupation, as indicated by body contraction and the initiation of pupal cuticle sclerotization. Data were recorded individually as the total number of days from hatching to pupal stage.

The caterpillar weight was obtained after 10 days of feeding on control and diets containing the inhibitors benzamidine, SKTI, and GORE3. Weights were utilized to calculate the mean and standard deviation for each experimental group. Pupal weight was evaluated as a developmental parameter following the larval stage.

Caterpillars surviving from each treatment were kept individually until pupation and subsequently weighed on an analytical balance 24 h after pupae formation. The weights were recorded, and the mean and standard deviation of pupal weight for each experimental group were subsequently calculated.

### 2.7 Nutritional Parameters

2.7

The analysis of nutritional parameters was based on caterpillar food consumption and body mass gain over a specified period. For this purpose, the following data were recorded: initial larval weight, final weight after 24 h feeding, weight of the provided diet, weight of the remaining diet, and weight of the feces. These data were used to calculate the following indices: efficiency of conversion of ingested food (ECI = (B/I) × 100); efficiency of conversion of digested food (ECD = (B/(I – F)) × 100); and approximate digestibility (AD = (I – F)/I) × 100), where B represents larval biomass gain (dry weight), I corresponds to the dry weight of food consumed, and F refers to the dry weight of feces excreted during the experimental period. A total of 15 larvae per treatment was used, for a total of 60 larvae. The caterpillars were individually placed in plastic vials containing 16 wells immediately after hatching. They were fed on artificial diet described in the previous sections until they reached the fourth instar. Subsequently, the diets were replaced with those containing the inhibitors at specified concentrations.

### 2.8 Consumed Leaf Area

For the leaf area experiment, Petri dishes (90 × 15 mm) containing 2% agar in water were prepared to prevent leaf desiccation. In each dish, maize (*Zea mays* L.) leaves were placed after being moistened with a water solution containing Tween 80 (0.01%) and the protease inhibitors GORE3, benzamidine, and SKTI, each at 0.1% (w/v). For the control group, only water with 0.01% Tween 80 was used.

Third-instar caterpillars were then individually introduced into each dish, for a total of 30 individuals per treatment. The insects remained in contact with the leaves 24 h to acclimate before the start of the evaluations. After this period, the leaves were replaced with those treated, and consumption was assessed cumulatively, that is, measured on the same leaf over time at 8, 24, and 48 h of caterpillar exposure. Before the start of the experiment, all leaves were photographed individually with an iPhone 13, and the procedure was repeated at each evaluation time point. The consumed leaf area was quantified using ImageJ 1.54g. Thirty replicates were performed for each treatment and control. Leaf area was measured from digital images at each evaluation time, enabling calculation of the consumed foliar area throughout the experimental period.^38^

### 2.9 Histopathology

Five alive caterpillars that reached to the sixth-instar from each treatment were dissected for histopathological analysis. The insects were dissected using microsurgical scissors, forceps, and pins. After removal of the midgut digestive system was transferred to Zamboni’s fixative solution^23^ for 24 h at 4 °C, washed twice in sodium phosphate buffer (PBS) 0.15 M, pH 7.2, and dehydrated through a graded ethanol series (70%, 80%, 90%, and 95%) for 10 min each. Subsequently, the midguts were embedded in historesin (Leica Biosystems Nussloch GmbH, Wetzlar, Germany) according to the manufacturer’s instructions. Sections of 3 μm thickness were obtained using a rotary microtome (Leica Biosystems Nussloch GmbH, Wetzlar, Germany). The sections were mounted on slides, stained with hematoxylin (15 min) and eosin (30 s), and subsequently analyzed and photographed using an Olympus BX-60 light microscope (Olympus Corporation, Tokyo, Japan).

### 2.10 Statistical Analysis

Trypsin activity, total protease activity, glutathione S-transferase (GST), catalase (CAT), and peroxidase (POX) were analyzed separately at each time point (24, 48, 72, and 96 h) using one-way ANOVA, with treatment as a fixed factor. When significant effects were detected (*p* < 0.05), means were compared with the control using Dunnett’s test. Assumptions of normality and homogeneity of variances were assessed using the Shapiro–Wilk and Levene tests, respectively.

Oxidative stress biomarkers (nitric oxide – NO, hydrogen peroxide – H_2_O_2_, malondialdehyde – MDA, protein carbonyl – PC, and superoxide dismutase – SOD) were analyzed using two-way ANOVA, considering treatment, inster, and their interaction as fixed factors. When significant effects were detected (*p* < 0.05), one-way ANOVAs were performed at each time point, followed by Dunnett’s test comparing treatments with the control. Assumptions of normality and homogeneity of variances were evaluated using the Shapiro–Wilk and Levene tests, respectively, and residuals were inspected to confirm model adequacy.

Insect survival was analyzed using the Kaplan–Meier estimator. Survival curves were compared using the log-rank (Mantel–Cox) test, and pairwise comparisons between treatments and the control were performed using Cox proportional hazards models to estimate hazard ratios (HR) and 95% confidence intervals.

Developmental parameters (pupal weight, larval period, and larval weight) were analyzed using one-way ANOVA followed by Tukey’s test. Assumptions of normality and homogeneity of variances were assessed using the Shapiro–Wilk and Levene tests, respectively. When heteroscedasticity was detected, Welch’s ANOVA followed by pairwise *t* tests with Benjamini–Hochberg (BH) adjustment was applied. For non-normal data with small sample sizes (*n* < 50), the Kruskal–Wallis test followed by pairwise Wilcoxon tests with BH correction was used.

Nutritional indices—Approximate Digestibility (AD), Efficiency of Conversion of Digested food (ECD), and Efficiency of Conversion of Ingested food (ECI)—were analyzed using one-way ANOVA, with treatment as a fixed factor. To account for heteroscedasticity, linear models with HC3 heteroscedasticity-consistent covariance estimators were also applied. Significant differences were evaluated using estimated marginal means (EMMs) followed by Tukey’s HSD test.

Cumulative leaf area consumption was analyzed using a linear mixed-effects model, with treatment, time, and their interaction as fixed effects, and experimental unit as a random effect. Fixed effects were assessed using Type III ANOVA with the Satterthwaite approximation, and model adequacy was verified through residual inspection.

All analyses were performed using R 4.5.2, with a significance level of 0.05 (α = 0.05).

## 3 Results

### 3.1 Molecular Dynamics Simulations, Structural Stability Analysis, and Binding Free-Energy Estimation

The structural dynamics of the *S. frugiperda* trypsin–inhibitor complexes were evaluated during 100 ns of molecular dynamics simulation using RMSD, RMSF, and hydrogen-bond analyses (Figure 1).

**Figure 1:**
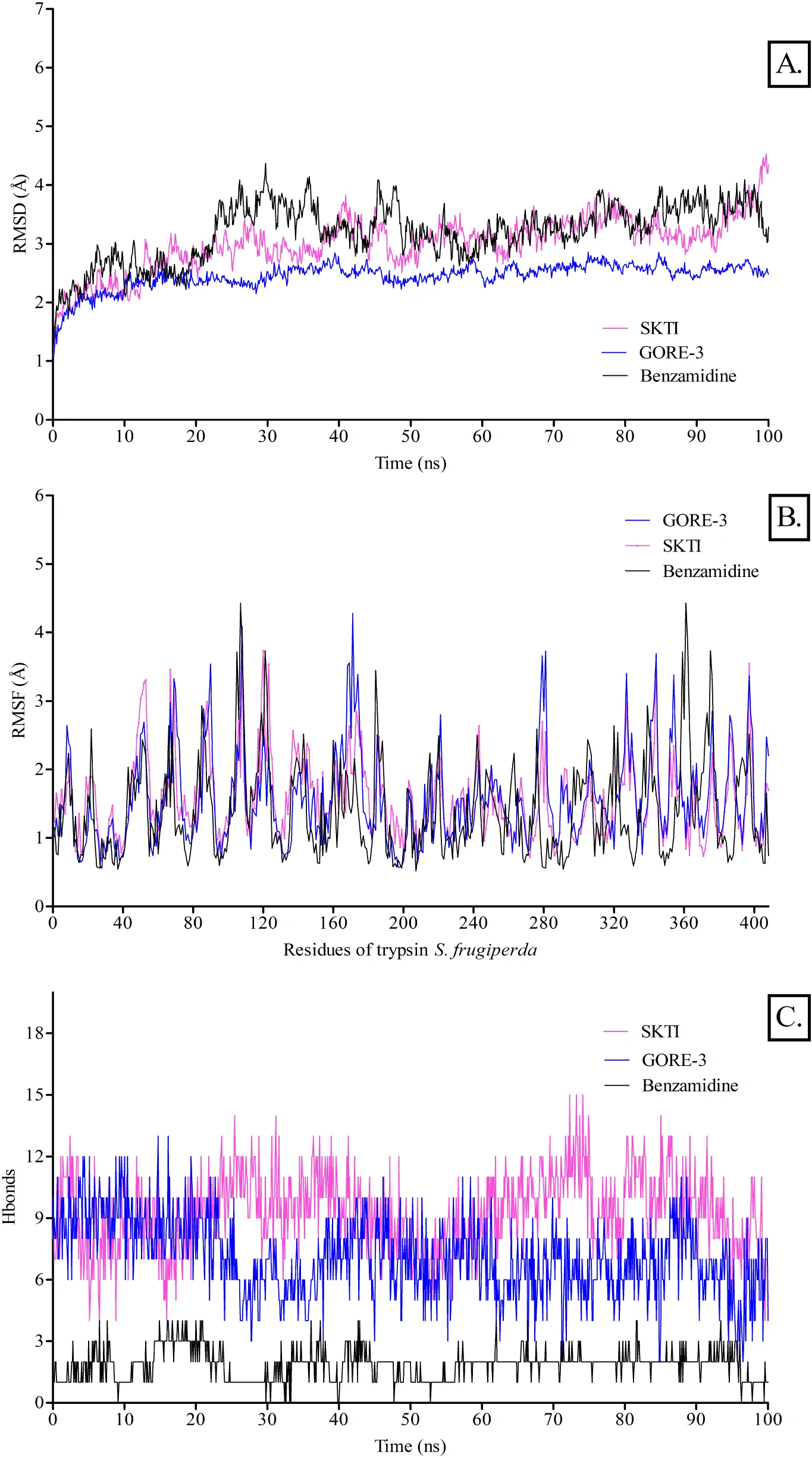
Structural stability and molecular interaction analysis of the complexes formed between *S. frugiperda* trypsin and the inhibitors SKTI, GORE3, and benzamidine during 100 ns of molecular dynamics simulation. A) Time evolution of the RMSD values of the complexes. B) Residue fluctuation profile evaluated by RMSF. C) Number of hydrogen bonds formed between trypsin and each inhibitor throughout the simulation.

The RMSD profiles showed distinct conformational behaviors among the three complexes (Figure 1A). The trypsin–GORE-3 complex exhibited the lowest and most stable RMSD profile throughout the simulation, remaining primarily between 2.2 and 2.8 Å after the initial equilibration period. In contrast, the trypsin–SKTI and trypsin–benzamidine complexes showed higher RMSD values and larger fluctuations. The trypsin–benzamidine complex fluctuated mainly between approximately 3.0 and 4.1 Å after 20 ns, whereas the trypsin–SKTI complex showed RMSD values mostly between 2.7 and 3.7 Å, with an increase toward the end of the simulation, reaching values above 4.0 Å.

The RMSF analysis revealed residue-dependent fluctuations along the trypsin sequence in all complexes (Figure 1B). Most residues exhibited RMSF values between approximately 0.6 and 2.5 Å. However, localized peaks were observed in specific regions of the enzyme, indicating higher mobility in those segments. The GORE-3 complex showed several pronounced peaks of fluctuation, particularly around residues near 170, 280, 330–360, and the C-terminal region. The benzamidine complex also showed localized regions of high fluctuation, including peaks near residues 110 and 360. The SKTI complex presented a comparable fluctuation profile, with several peaks distributed along the enzyme sequence, although most residues remained below 3.0 Å.

Hydrogen-bond analysis showed clear differences in the number of intermolecular hydrogen bonds maintained during the simulation (Figure 1C). The trypsin–SKTI complex presented the highest number of hydrogen bonds, with values generally ranging from approximately 6 to 15 throughout the trajectory. The trypsin–GORE-3 complex showed an intermediate hydrogen-bond profile, with approximately 4–10 hydrogen bonds, with moderate fluctuations over time. In contrast, the trypsin–benzamidine complex showed the fewest hydrogen bonds, generally ranging from 0 to 4 during the simulation.

Entropy-uncorrected MM/GBSA and MM/PBSA effective interaction-energy estimates were calculated for the trypsin–SKTI, trypsin–benzamidine, and trypsin–GORE3 complexes. The energetic components are presented as mean ± standard deviation in Table 1. These values were used as qualitative energetic descriptors of the simulated complexes and were not interpreted as absolute binding affinities.

**Table 1:** Entropy-Uncorrected MM/GBSA and MM/PBSA Effective Interaction-Energy Estimates and Energetic Components of the *S. Frugiperda* trypsin–inhibitor Complexes^a^

*Complex*	*Method*	*Δ*_*VDWAALS*_	*Δ*_*EEL*_	*Δ*_*EGB*_ */ Δ*_*EPB*_	*Δ*_*ESurf*_ */ Δ*_*ENpolar*_	*ΔG*_*SOLV*_	*ΔG*_*Binding*_
*Trypsin–SKTI*	GBSA	-75.04 ± 13.51	-299.18 ± 34.59	343.83 ± 29.85	-11.15 ± 1.55	332.68 ± 30.07	-41.54 ± 10.23
*Trypsin–SKTI*	PBSA	-75.04 ± 13.51	-299.18 ± 34.59	329.58 ± 27.98	-9.65 ± 0.91	319.92 ± 28.02	-54.30 ± 9.42
*Trypsin–Benzamidine*	GBSA	-49.37 ± 4.61	-91.01 ± 20.60	119.26 ± 20.54	-6.41 ± 0.71	112.85 ± 20.09	-27.52 ± 5.18
*Trypsin–Benzamidine*	PBSA	-49.37 ± 4.61	-91.01 ± 20.60	119.21 ± 22.28	-5.18 ± 0.39	114.03 ± 22.01	-26.35 ± 7.83
*Trypsin–GORE3*	GBSA	-64.04 ± 8.15	-245.16 ± 25.53	225.66 ± 18.23	-10.13 ± 1.12	232.45 ± 20.45	-38.12 ± 8.13
*Trypsin–GORE3*	PBSA	-64.04 ± 8.15	-245.16 ± 25.53	222.36 ± 12.31	-9.22 ± 1.23	214.28 ± 22.80	-42.30 ± 7.35

a Values are expressed as mean ± standard deviation in Kcal/Mol. Δ_VDWAALS_: van der Waals contribution; Δ_EEL_: electrostatic contribution; Δ_EGB_/Δ_EPB_: polar solvation energy estimated by GBSA or PBSA; Δ_ESURF_/Δ_ENPOLAR_: nonpolar solvation contribution; Δ_GSOLV_: total solvation energy; ΔG_Binding_: entropy-uncorrected effective interaction-energy estimate. These values were used only as relative descriptors within the same computational protocol and were not interpreted as absolute binding affinities or as strict quantitative rankings across chemically distinct inhibitors.

For the trypsin–SKTI complex, the entropy-uncorrected MM/GBSA and MM/PBSA effective interaction-energy estimates were −41.54 ± 10.23 kcal/mol and −54.30 ± 9.42 kcal/mol, respectively. The van der Waals contribution was −75.04 ± 13.51 kcal/mol in both methods, whereas the electrostatic contribution was −299.18 ± 34.59 kcal/mol. The polar solvation term was 343.83 ± 29.85 kcal/mol for GBSA and 329.58 ± 27.98 kcal/mol for PBSA. The nonpolar solvation contribution was −11.15 ± 1.55 kcal/mol for GBSA and −9.65 ± 0.91 kcal/mol for PBSA (Table 1).

The trypsin–benzamidine complex presented entropy-uncorrected MM/GBSA and MM/PBSA effective interaction-energy estimates of −27.52 ± 5.18 kcal/mol and −26.35 ± 7.83 kcal/mol, respectively. The van der Waals contribution was −49.37 ± 4.61 kcal/mol, and the electrostatic contribution was −91.01 ± 20.60 kcal/mol in both methods. The polar solvation term was 119.26 ± 20.54 kcal/mol for GBSA and 119.21 ± 22.28 kcal/mol for PBSA, whereas the nonpolar solvation contribution was −6.41 ± 0.71 kcal/mol and −5.18 ± 0.39 kcal/mol, respectively (Table 1).

For the trypsin–GORE3 complex, the entropy-uncorrected MM/GBSA and MM/PBSA effective interaction-energy estimates were −38.12 ± 8.13 kcal/mol and −42.30 ± 7.35 kcal/mol, respectively. The van der Waals contribution was −64.04 ± 8.15 kcal/mol, and the electrostatic contribution was −245.16 ± 25.53 kcal/mol in both methods. The polar solvation contribution was 225.66 ± 18.23 kcal/mol for GBSA and 222.36 ± 12.31 kcal/mol for PBSA. The nonpolar solvation contribution was −10.13 ± 1.12 kcal/mol for GBSA and −9.22 ± 1.23 kcal/mol for PBSA (Table 1).

Although the MM/GBSA and MM/PBSA estimates should not be interpreted as absolute binding affinities, the energetic decomposition provides useful qualitative information about the interaction patterns of the simulated complexes. For GORE3, both van der Waals and electrostatic terms contributed favorably to the entropy-uncorrected effective interaction-energy estimate, while the positive polar solvation component partially counterbalanced these favorable gas-phase interactions. This pattern suggests that the trypsin–GORE3 complex is supported by a combination of short-range contacts and electrostatic interactions, rather than by a single dominant energetic component.

The MM/GBSA and MM/PBSA calculations were therefore used as entropy-uncorrected effective interaction-energy descriptors under the same computational protocol. Because SKTI, GORE3, and benzamidine differ markedly in molecular size, chemical nature, number of interacting atoms, conformational flexibility, and binding interface, the calculated values were not interpreted as absolute binding affinities or as strict quantitative rankings among inhibitors. Accordingly, although GORE3 showed more negative van der Waals and electrostatic contributions than benzamidine under the applied protocol, this difference was interpreted only as a qualitative descriptor of interaction behavior and not as evidence of stronger experimental affinity.

The calculated profiles showed negative van der Waals and electrostatic contributions for all complexes, whereas the polar solvation terms were positive and partly compensated the favorable gas-phase interactions. This compensation was particularly evident for the trypsin–SKTI complex, which showed a large favorable electrostatic term counterbalanced by a large unfavorable polar solvation contribution. Therefore, the energetic decomposition was interpreted qualitatively, especially for charged or highly polar interfaces, where continuum-solvent end-point methods are known to be sensitive to electrostatic and solvation approximations.

The relatively large standard deviations observed among snapshots indicate conformational variability in the calculated energetic estimates. Therefore, differences between complexes, particularly between SKTI and GORE3, should not be overinterpreted as statistically resolved differences in binding affinity. The MM/GBSA and MM/PBSA results were thus used to support the molecular dynamics analyses as qualitative energetic descriptors, rather than as independent predictors of experimental inhibitory potency.

### 3.2 Persistence of Trypsin-Like Enzyme and Total-Proteases Inhibition by Peptides in the Midgut of *S. Frugiperda*

Trypsin activity was influenced by treatments, time points, and their interaction, indicating that treatment effects varied over time. At 24 h, the control caterpillars showed higher activity than benzamidine (p = 0.000058), GORE3 (p = 0.00201), and SKTI (p = 0.00275). GORE3 and SKTI also showed higher activity than benzamidine (p = 0.0222 and p = 0.0154, respectively), with no difference between them (p = 0.9923). At 48 h, the control showed higher activity than benzamidine (*p* < 0.001), GORE3 (p = 0.000109), and SKTI (*p* < 0.001). GORE3 showed higher activity than benzamidine (p = 0.000003) and SKTI (p = 0.000001), while Benzamidine and SKTI did not differ from each other (p = 0.309). At 72 h, GORE3 showed significantly higher activity than all other treatments (*p* < 0.001 in all comparisons). The control showed higher activity than benzamidine (p = 0.000207) and SKTI (p = 0.000122), while Benzamidine and SKTI did not differ (p = 0.926). At 96 h, SKTI showed the highest activity, differing from Benzamidine (*p* < 0.001), Control (p = 0.000586), and GORE3 (*p* < 0.001). The control showed higher activity than benzamidine (*p* < 0.001) and GORE3 (p = 0.000002). GORE3 showed higher activity than benzamidine (p = 0.000001). (Figure 2A).

**Figure 2:**
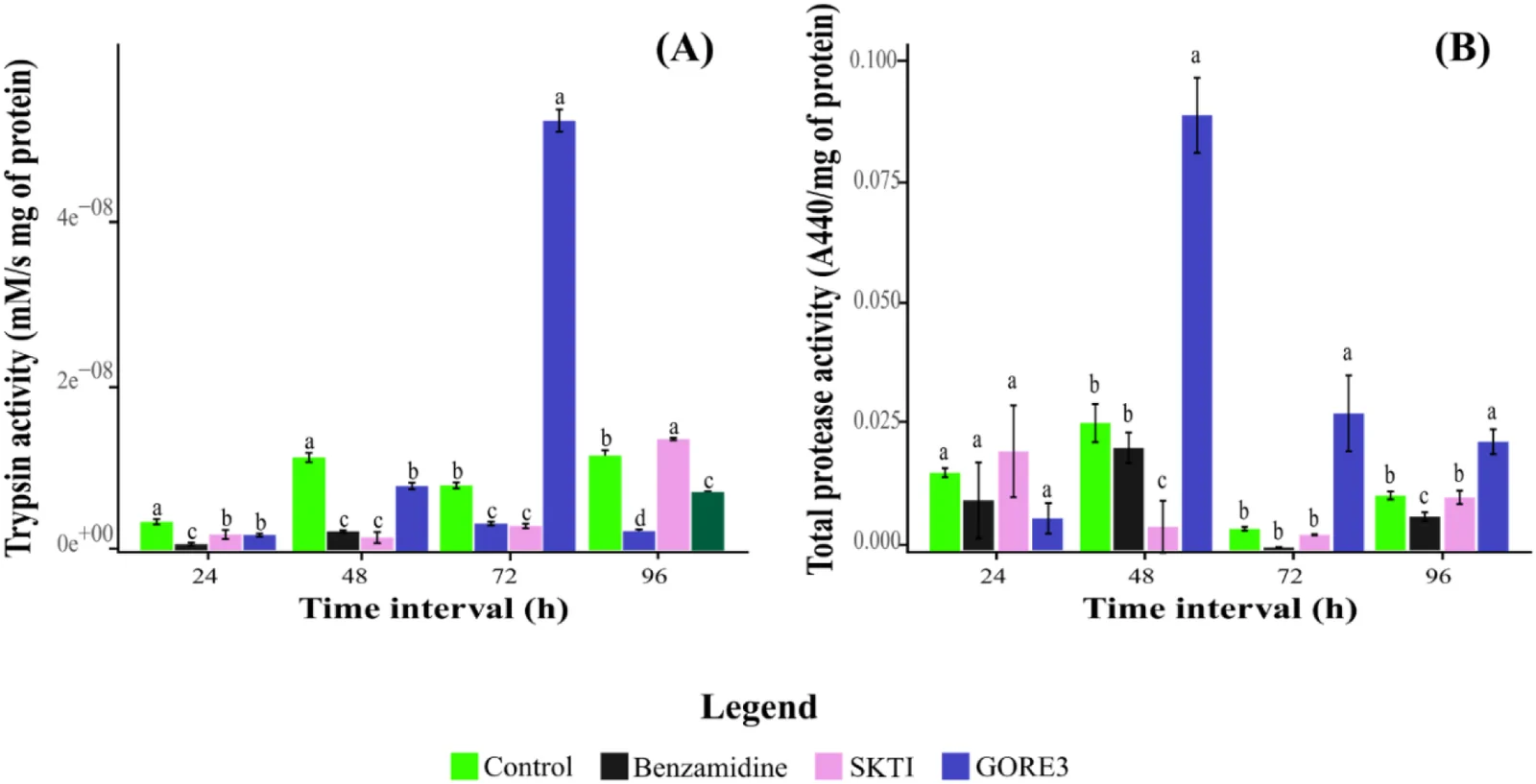
Persistence of peptides GORE3, benzamide, and SKTI inhibition in trypsins (Figure 2A) and total proteolytic activity. (Figure 2B). Bars indicate mean ± SE; *n* = 10). The bars above indicate statistical differences by Dunnett’s test (*p* < 0.05).

Protease activity varied significantly according to treatment and time, with a significant interaction between factors (two-way ANOVA: treatment, *p* < 2 × 10^–1 6^; instar, *p* < 2 × 10^–1 6^; interaction, *p* < 2 × 10^–1 6^). At 24 h, GORE3 exhibited the highest protease activity, differing significantly from Benzamidine (*p* < 0.01) and the control (*p* < 0.05), while SKTI showed intermediate values and did not differ from these treatments (*p* > 0.05). At 48 h, the same pattern was observed, with GORE3 maintaining higher activity compared to the Control (*p* < 0.05). In contrast, benzamidine and SKTI showed intermediate responses and did not differ significantly from the other treatments (*p* > 0.05). At 72 h, the differences among treatments became more pronounced. GORE3 showed significantly higher protease activity than all other treatments (all *p* < 0.01), whereas Benzamidine, Control, and SKTI did not differ from each other (all *p* > 0.05). At 96 h, this pattern was maintained, with GORE3 consistently presenting the highest activity, differing from Benzamidine (*p* < 0.01), Control (*p* < 0.01), and SKTI (*p* < 0.01), while these three treatments remained statistically similar (all *p* > 0.05). (Figure 2B).

### 3.3 Oxidative Stress

For total antioxidant capacity, there was a significant effect of treatment (F = 6.117; p = 0.0022) and instar (F = 22.151; p = 1.06 × 10^–6^), with no significant interaction between these factors (F = 1.971; p = 0.100). Differences among treatments occurred in third instar caterpillars (F = 4.215; p = 0.0238) and fifth instar (F = 15.42; p = 0.0011), with higher values associated with GORE3 in both instars, whereas in fourth instar, no significant difference was observed (F = 2.85; p = 0.105). (Figure 3A).

**Figure 3:**
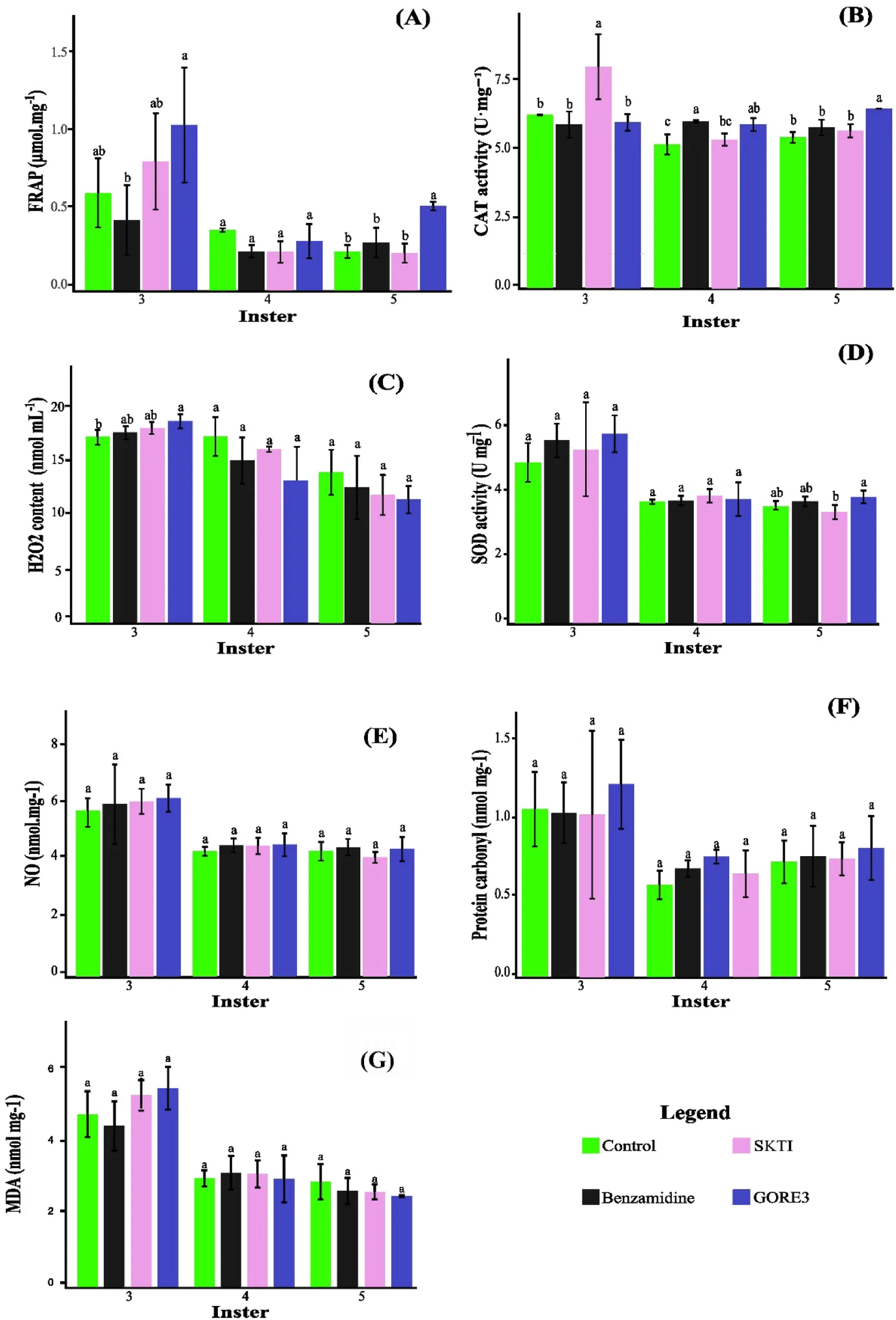
Oxidative stress biomarkers and antioxidant enzyme activities in *Spodoptera frugiperda* larvae exposed to protease inhibitors. Treatments consisted of Control, Benzamidine, SKTI, and GORE3. (A) Ferric reducing antioxidant power (FRAP); (B) Catalase (CAT) activity; (C) Hydrogen peroxide (H_2_O_2_) content; (D) Superoxide dismutase (SOD) activity; (E) Nitric oxide (NO) levels; (F) Protein carbonyl content; and (G) Lipid peroxidation measured as malondialdehyde (MDA). Bars represent the mean ± SE. Different letters indicate significant differences among treatments within each larval instar according to Dunnett’s test (*p* < 0.05).

Catalase activity was influenced by treatment (F = 4.955; p = 0.00811), instar (F = 15.797; p = 4.19 × 10^–5^), and by the interaction between these factors (F = 8.784; p = 4.06 × 10^–5^). Due to the significant interaction, comparisons among treatments were performed within each instar. At the third instar, a significant effect of treatment was observed (F = 6.749; p = 0.0139), with higher activity in SKTI compared to Benzamidine (p = 0.019), Control (p = 0.045), and GORE3 (p = 0.024). In contrast, the latter treatments did not differ from each other. At the fourth instar, treatment also affected catalase activity (F = 8.346; p = 0.00759). Benzamidine showed higher activity than Control (p = 0.0119) and SKTI (p = 0.047), while GORE3 was higher than Control (p = 0.027). No differences were observed among the remaining comparisons. At the fifth instar, a significant treatment effect was again detected (F = 13.74; p = 0.0016), with GORE3 showing higher activity than Benzamidine (p = 0.016), Control (p = 0.0013), and SKTI (p = 0.0068). In contrast, the other treatments did not differ from each other. (Figure 3B).

Hydrogen peroxide levels were influenced by larval instar (F = 40.747; p = 1.6 × 10^–9^), with no significant effect of treatment (F = 0.948; p = 0.4289) and no interaction between treatment and instar (F = 2.199; p = 0.0689). In the analysis by instar, differences among treatments were observed only in instar 3 (F = 4.867; p = 0.0136), where GORE3 showed higher values than the control (p = 0.0098), whereas Benzamidine and SKTI did not differ from the other treatments. In the fourth (F = 1.595; p = 0.265) and fifth instars (F = 0.824; p = 0.517), no significant differences among treatments were detected. (Figure 3C).

SOD activity was influenced only by instar (F = 36.561; p = 6.99 × 10^–9^), with no effect of treatment (F = 1.149; p = 0.345) and no interaction between factors (F = 0.412; p = 0.865). Accordingly, no differences among treatments were observed within any instar. At the third instar, treatment had no effect on SOD activity (F = 0.898; p = 0.465), and all treatments showed similar values. At the fourth instar, no treatment effect was detected (F = 0.226; p = 0.876), and there were no differences among treatments. At the fifth instar, the treatment effect was marginal (F = 3.879; p = 0.0556). Overall, no consistent differences among treatments were observed, although SKTI differed slightly from GORE3 in a pairwise comparison (p = 0.0485). (Figure 3D).

Nitric oxide production was influenced only by instar (F = 35.496; p = 9.62 × 10^–9^), with no effect of treatment (F = 0.505; p = 0.682) and no interaction (F = 0.160; p = 0.986), and no differences among treatments were observed at any instar. (Figure 3E).

Carbonylated proteins also showed a significant effect only of instar (F = 11.739; p = 0.00015), with no treatment effect (F = 0.746; p = 0.533) and no interaction (F = 0.084; p = 0.997). Similarly, SOD activity was influenced exclusively by instar (F = 36.561; p = 6.99 × 10^–9^), with no treatment effect (F = 1.149; p = 0.345) and no interaction (F = 0.412; p = 0.865). (Figure 3F).

MDA levels were influenced only by instar (p = 2.86 × 10^–1 3^), with no effect of treatment (p = 0.199) and no interaction between factors (p = 0.167). No differences among treatments were observed within the third instar (p = 0.089), fourth instar (p = 0.954), or fifth instar (p = 0.505). Accordingly, Tukey’s post hoc comparisons showed no significant differences among treatments at any instar (Figure 3G).

### 3.4 Survival Analysis

Larval survival differed among treatments (p = 0.00031). Compared with the control, GORE3 significantly increased the risk of death over time (p = 0.00185). Benzamidine showed a marginal increase in risk (p = 0.0675), while SKTI did not differ from the control caterpillars (p = 0.6563). These results indicate that only GORE3 significantly reduced larval survival compared to the control group. (Figure 4).

**Figure 4:**
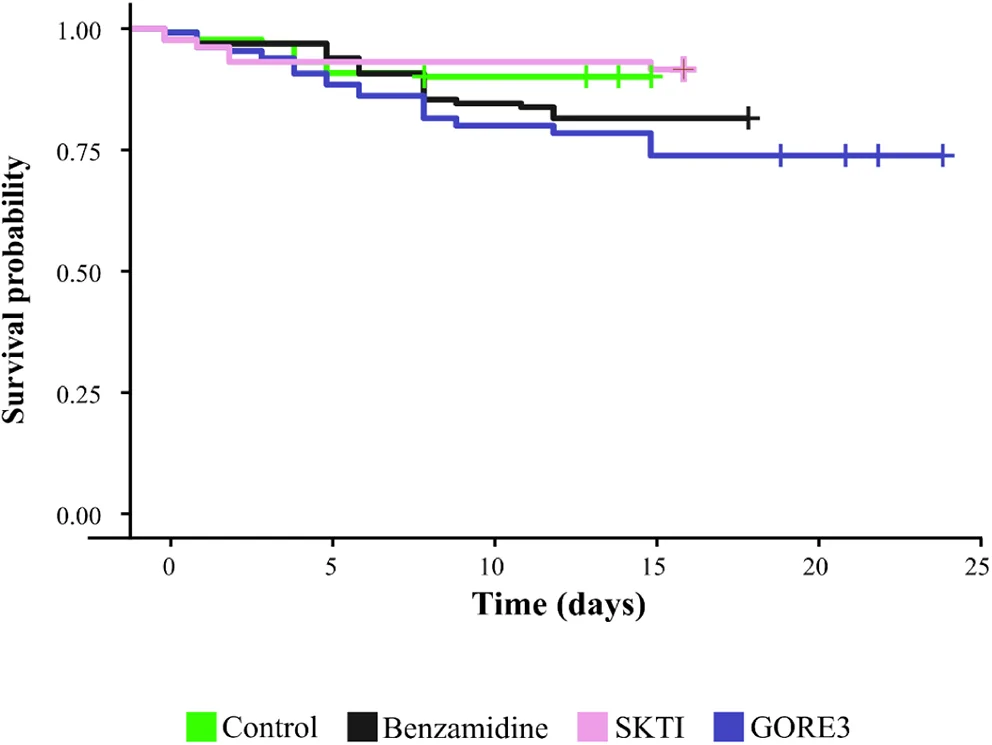
Survival plots of *S. frugiperda* (Lepidoptera: Noctuidae) larvae reared on a diet containing GORE3 peptide, benzamidine and SKTI (positive control) inhibitors at a concentration of 0.1% mass of the inhibitor per 100 mL of liquid diet. A diet without inhibitors was used as the negative control.

### 3.5 Biological Cycle and Body Mass of Larvae and Pupae

The total larval period was affected by treatments (F_3_,_254_,_12_ = 58.80; *p* < 0.001). The control caterpillars showed a shorter duration than those exposed to benzamidine and GORE3 (both *p* < 0.001), whereas SKTI showed intermediate values, distinct from the others. There was no significant difference between the total period in caterpillars fed on Benzamidine and GORE3. (Figure 5A).

**Figure 5:**
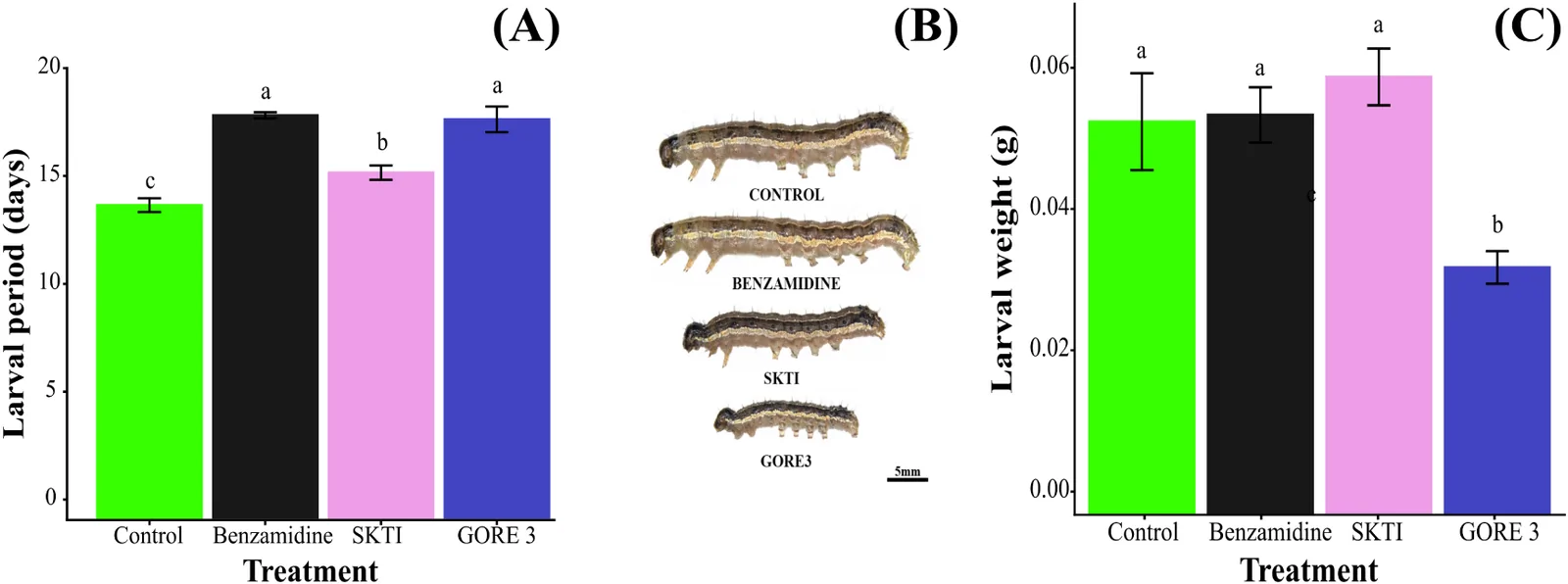
Effects of protease inhibitors on *S. frugiperda* development. (Figure 5A) Larval period. (Figure 5B) Representative larvae showing differences in body size among treatments. (Figure 5C) Larval weight. Values represent mean ± SE (*n* = 130) for control, benzamidine, SKTI, and GORE3. Different letters indicate significant differences among treatments (*p* < 0.05).

Caterpillar weight was influenced by treatments (F_3_,_275_,_36_ = 15.85; *p* < 0.001). The GORE3 treatment resulted in lower larval weight compared to the control (p = 0.0033), whereas Benzamidine (p = 0.0029)- and SKTI (p = 0.00045)-treated caterpillars were similar to the control. (Figure 5C).

Pupal weight differed among treatments (F_3_,_137_ = 4.16; p = 0.0075). Pupae from the SKTI treatment had a lower weight than those from the control (p = 0.0418), whereas those from caterpillars fed on GORE3 (p = 0.0269) were similar. Benzamidine did not differ from the control (p = 0.5548), GORE3 (p = 0.5646), or SKTI (p = 0.3748; Figures 6 and 7).

**Figure 6:**
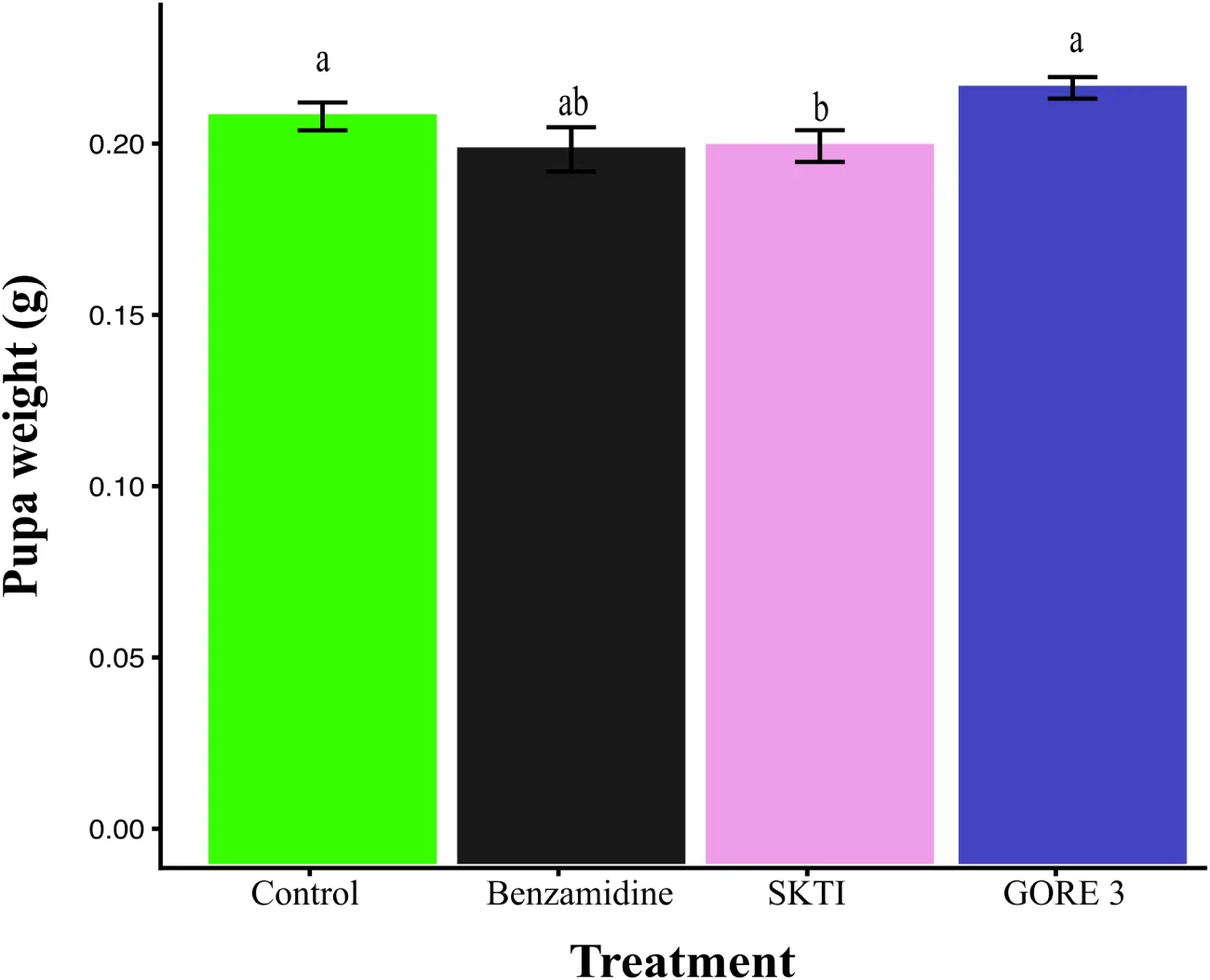
Effects of protease inhibitors on pupal weight of *Spodoptera frugiperda*. Bars represent mean ± SE for Control, benzamidine, SKTI, and GORE3 (0.1% w/v). Different letters above the bars indicate significant differences among treatments according to Dunnett’s test (*p* < 0.05).

**Figure 7:**
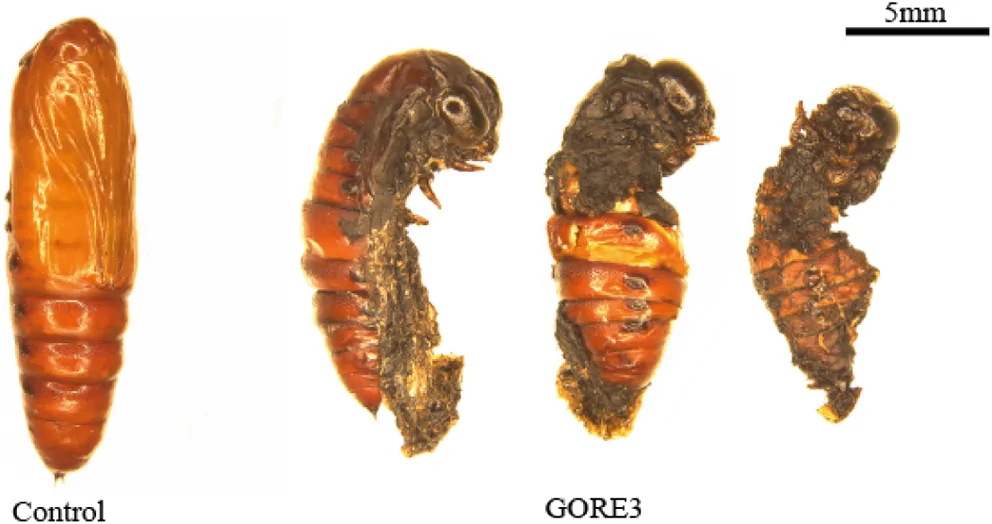
Representative pupae of *S. frugiperda* under different treatments. The control group exhibited intact and normally developed pupae, whereas individuals exposed to GORE3 (0.1% w/v) showed severe morphological alterations, including body deformation, incomplete pupation, and tissue deterioration. Scale bar = 5 mm.

### 3.6. Nutritional Parameters

3.6

Treatments significantly influenced nutritional indices. Approximate digestibility (AD) differed among groups (F_3_,_54_ = 7.99, *p* < 0.001), with higher values observed in caterpillars fed on benzamidine and GORE3 than in the control. SKTI was similar to that of control caterpillars (Figure 8A).

**Figure 8:**
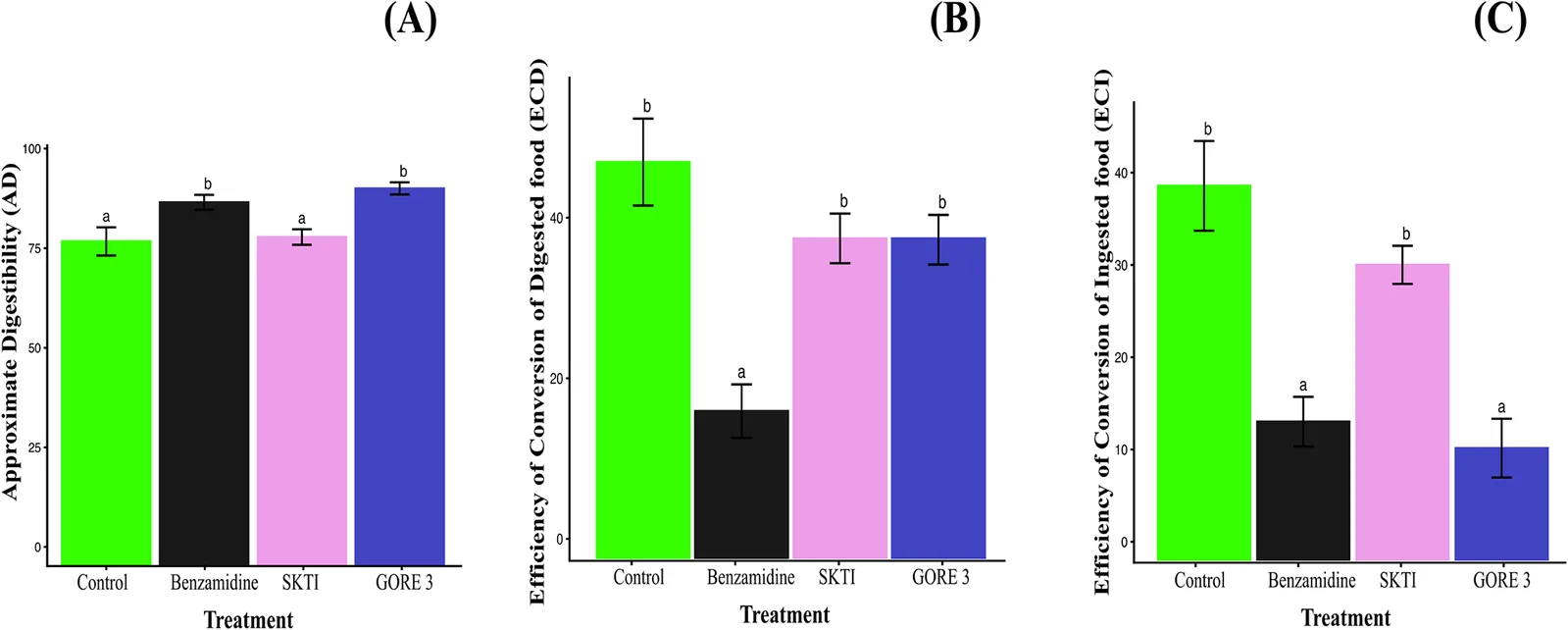
Effects of protease inhibitors on nutritional indices of *S. frugiperda*. (A) Approximate digestibility (AD), (B) efficiency of conversion of digested food (ECD) and (C) efficiency of conversion of ingested food (ECI). Bars represent mean ± SE (*n* = 30 larvae per treatment) for Control, benzamidine, SKTI, and GORE3 (0.1% w/v). Different letters above the bars indicate significant differences among treatments according to Dunnett’s test (*p* < 0.05).

For the efficiency of conversion of digested food (ECD), there was a significant treatment effect (F_3_,_53_ = 11.01, *p* < 0.001). Benzamidine showed a significantly lower value than the control, whereas GORE3 and SKTI did not differ and formed a group with higher efficiency (Figure 8B). Similarly, the efficiency of conversion of ingested food (ECI) was also affected by treatments (F_3_,_54_ = 16.66, *p* < 0.001), forming two distinct groups: GORE3 and benzamidine showed the lower values than SKTI, which was similar to the control (Figure 8C).

### 3.7 Consumed Leaf Area

Leaf area consumption varied significantly over time, whereas no significant effects of treatment or the interaction between treatment and time were observed (linear mixed-effects model). The factor time had a significant effect on leaf area consumption (F_3_,_348_ = 3.61, p = 0.0136), indicating progressive cumulative consumption throughout the evaluation period. In contrast, treatments did not differ significantly (F_3_,_116_ = 0.84, p = 0.4742), and the interaction between treatment and time was not significant (F_9_,_348_ = 0.27, p = 0.9823), indicating that the temporal pattern of leaf consumption was similar among treatments. The cumulative leaf area consumed increased gradually from 0 to 48 h in all treatments, with overlapping standard errors throughout the experiment, reinforcing the absence of treatment effects on feeding behavior (Figure 9).

**Figure 9:**
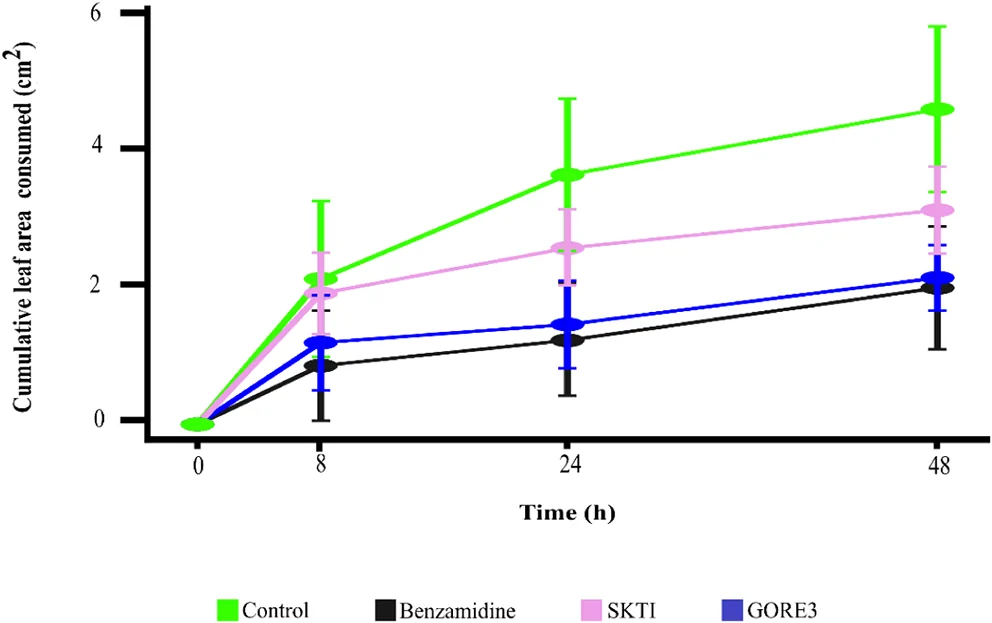
Cumulative leaf area consumed over time by *Spodoptera frugiperda* under different treatments. Points represent the mean values (*n* = 30 per treatment) of cumulative consumption at each evaluation interval (0h, 8h, 24h, and 48 h).

### 3.8 Histology

The midgut of sixth-instar *S. frugiperda* caterpillars from the control group exhibited a morphology consistent with its developmental stage (Figure 10A).

**Figure 10:**
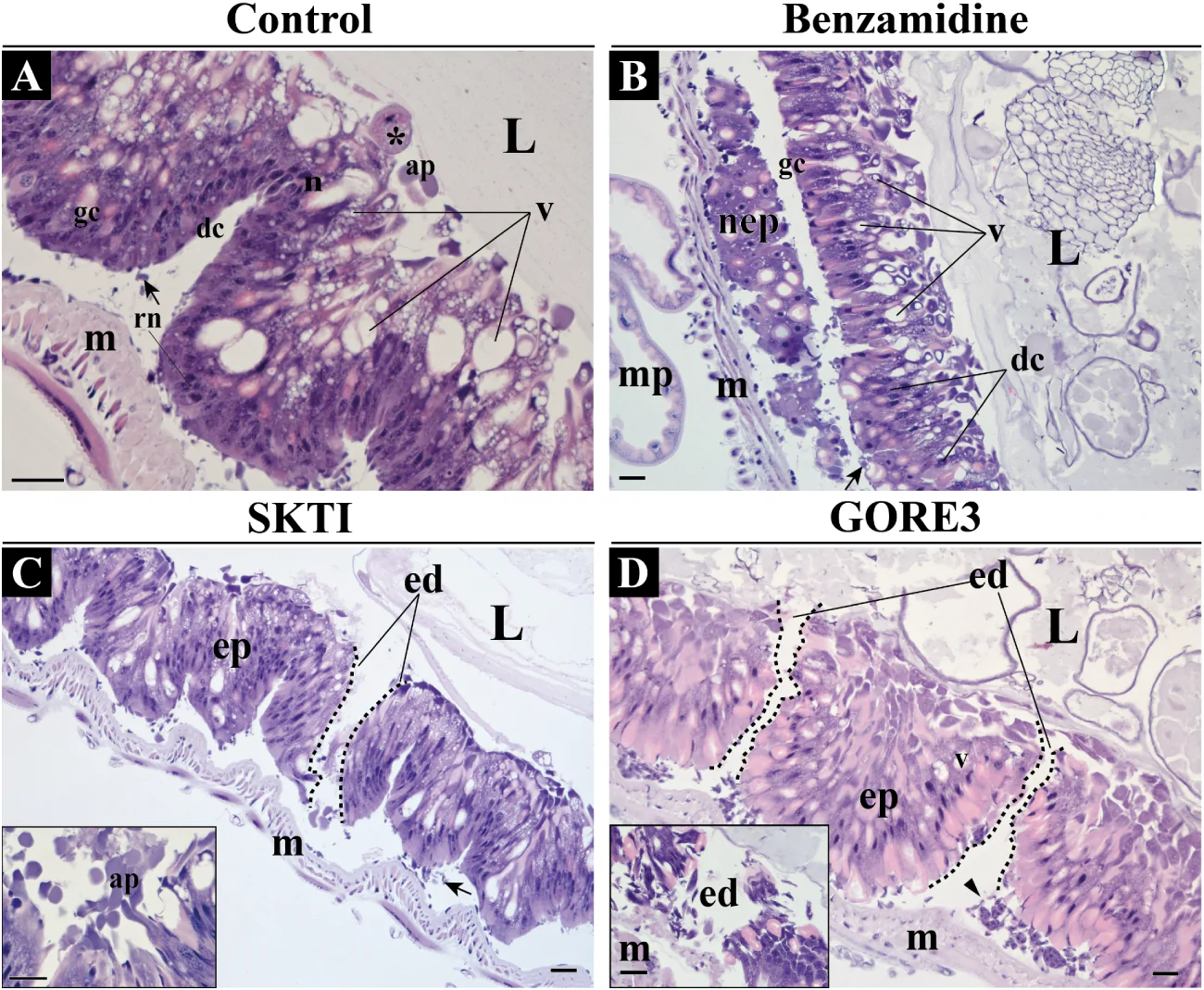
Micrographs of the midgut of sixth-instar *Spodoptera frugiperda* larvae under different treatments. (A) Control group showing typical epithelial organization with digestive cells (dc), goblet cells (gc), and regenerative cell nests (rn). Cell fragments (asterisks) are observed within the lumen (L). Note the apical vacuolization (v) and the presence of basophilic structures (arrows) near the muscle layer (m). (B) Benzamidine treatment. Individuals exhibiting a conformation characteristic of an advanced stage (metamorphosis), with the formation of the new epithelium (nep) and vacuolization (v). Malpighian tubules (mp) are observed adjacent to the organ. (C) SKTI treatment. Histopathological changes characterized by epithelial disruptions (ed, delimited by dashed lines) and apocrine secretions (ap). Basophilic structures (arrows) present in the basal portion. (D) GORE3 treatment. Epithelial disruptions (ed) with loss of adhesion between cell morphotypes. The epithelium (ep) is unstructured, with cells migrating toward the basal region (arrowhead). The muscle layer (m) is discontinuous and disorganized. Lumen (L); nucleus (n). epithelium (ep). Scale bars: 25 μm.

The midgut epithelium displays typical cell morphotypes, including digestive cells and goblet cells, both with apical vacuolization, as well as scarce regenerative cell nests (Figure 10A). Small basophilic bodies and scattered cell nuclei were observed between the epithelium and the muscle layer; the latter is detached from the epithelial portion and features a transverse layer composed of two strata. Additionally, the release of cell fragments into the lumen is visible (Figure 10A).

Individuals treated with benzamidine presented a conformation characteristic of an advanced developmental stage. The midgut showed an organization consisting of two cell layers: the larval epithelium in the apical portion, which already exhibited high vacuolization and cellular condensation (Figure 10B), and portions of the new midgut epithelium that will arise during the metamorphic process. The muscle layer is positioned close to the epithelium.

The caterpillars exposed to SKTI showed midgut histopathological alterations, including epithelial disruptions characterized by discontinuous areas and loss of epithelial integrity, as well as apocrine secretions (Figure 10C inset). Nonetheless, they maintained the morphotypic configuration of digestive and goblet cells. Furthermore, the absence of distinct regenerative cell nests and the presence of basophilic structures in the basal portion were observed (Figure 10C). The muscle layer is widely separated from the epithelium, with a duplicated transverse cell layer.

The GORE3 treatment induced histopathological changes in the midgut. Epithelial disruptions resulted in a loss of cell adhesion and architecture (Figure 10D). In regions with some epithelial integrity (Figure 10D), the muscle layer is noted to possess two transverse strata and one longitudinal stratum. However, in the space between the epithelium and the muscle layer, several cells appear to migrate toward the basal portion of the organ (Figure 10D). In damaged areas (Figure 10D inset), cells lose contact and their typical morphology, becoming slender and obscuring the distinction between morphotypes. The muscle layer also appeared discontinuous, with separation between cells and strata.

## 4 Discussion

This study provides an integrated view of how the synthetic peptide GORE3 affects the digestive physiology of *S. frugiperda* by connecting molecular interaction profiles with enzymatic regulation, larval performance, and midgut tissue integrity. The results suggest that GORE3 should not be interpreted only as a trypsin-like enzyme inhibitor, but as a peptide capable of triggering a broader physiological response in larvae. This response included altered protease activity, impaired larval performance, reduced survival, and severe histopathological changes in the midgut. The comparison among benzamidine, SKTI, and GORE3 is particularly important because these inhibitors represent distinct biochemical categories: benzamidine is a small, competitive inhibitor; SKTI is a proteinaceous, plant-derived inhibitor; and GORE3 is a synthetic peptide. Therefore, the differences observed among treatments provide a useful framework for understanding how inhibitor structure and interaction profiles may influence biological outcomes.

Previous work showed that GORE3 interacts with *S. frugiperda* trypsin-like proteases, occupies the catalytic cleft, and acts as a competitive inhibitor, affecting enzymatic activity and larval development.^12^ The present molecular dynamics analysis extends those findings by showing that the trypsin–GORE3 complex remained structurally stable during 100 ns of simulation. Among the three evaluated complexes, trypsin–GORE3 displayed the lowest and most stable RMSD profile, suggesting that the complex underwent limited global conformational deviation after equilibration. This is a relevant observation because GORE3 is smaller than SKTI yet maintains a stable association with the enzyme throughout the trajectory. In contrast, trypsin–SKTI and trypsin–benzamidine showed higher RMSD values and larger fluctuations, indicating more pronounced conformational rearrangements during the simulation.

The stability of the trypsin–GORE3 complex is especially meaningful when considered together with the peptide nature of GORE3. Unlike benzamidine, which is a small molecule mainly associated with active-site recognition, GORE3 may interact with a broader region of the catalytic cleft and adjacent subsites.^39^ At the same time, unlike SKTI, GORE3 does not depend on a large protein–protein interface to maintain its interaction with trypsin.^17,40^ Therefore, the low RMSD profile observed for trypsin–GORE3 suggests that this peptide likely strikes a balance between structural compactness and sustained interactions, thereby maintaining dynamic stability within the enzymatic environment.

The RMSF profiles showed that all complexes retained residue-dependent flexibility, with localized peaks in specific regions of the enzyme.^30,41^ In the GORE3 complex, the coexistence of low RMSD values with localized RMSF peaks suggests that the global structure of trypsin was preserved while specific regions remained flexible.^17^ This pattern does not indicate generalized destabilization of the enzyme. Instead, it suggests that GORE3 binding allows local conformational adjustments while maintaining the complex’s overall stability. Such behavior is relevant to digestive proteases because ligand binding does not necessarily require complete rigidification of the enzyme; stable binding may occur alongside local mobility in loops or peripheral regions.^11^

Hydrogen-bond analysis further differentiated the interaction profile of GORE3 from those of SKTI and benzamidine. SKTI formed the most hydrogen bonds throughout the simulation, consistent with its proteinaceous nature and larger surface area for interactions.^40^ Benzamidine showed the fewest hydrogen bonds, consistent with its smaller size and more restricted interaction pattern. GORE3 displayed an intermediate but persistent hydrogen-bond profile, indicating that its interaction with trypsin was less extensive than that of SKTI but more sustained than that of benzamidine. This result is important because it shows that the biological relevance of GORE3 is not necessarily associated with the highest number of hydrogen bonds, but rather with a stable and persistent interaction network sufficient to maintain the peptide within the binding region.^17^

The interpretation of the MM/GBSA and MM/PBSA results requires caution. These end-point methods provide approximate, entropy-uncorrected effective energetic estimates and are sensitive to ligand size, charge distribution, conformational flexibility, and the extent of the binding interface.^17,41^ Therefore, direct quantitative comparisons among SKTI, GORE3, and benzamidine are limited because these inhibitors represent distinct molecular classes: a proteinaceous inhibitor, a synthetic peptide, and a small molecule, respectively. In this context, the calculated values should not be interpreted as experimental binding affinities or as direct potency rankings among inhibitors.

In the present study, the MM/GBSA and MM/PBSA estimates were used as qualitative energetic descriptors obtained under the same computational protocol, rather than as absolute binding free energies. The energetic decomposition showed negative electrostatic and van der Waals contributions for the evaluated complexes, together with positive polar solvation terms that partly compensated favorable gas-phase interactions. For GORE3, both van der Waals and electrostatic terms contributed favorably to the entropy-uncorrected effective interaction-energy estimate, suggesting that the trypsin–GORE3 complex is supported by a combination of short-range contacts and electrostatic interactions, rather than by a single dominant energetic component. This interpretation is consistent with the relevance of local interaction networks and residue-level recognition in serine protease–ligand complexes.^42,43^

This compensation between favorable gas-phase interactions and unfavorable polar solvation was especially evident for the trypsin–SKTI complex, which showed a large favorable electrostatic term counterbalanced by a large unfavorable polar solvation contribution. Therefore, these values were interpreted qualitatively, particularly for charged or highly polar interfaces, where continuum-solvent end-point approaches are sensitive to electrostatic and solvation approximations.

This limitation is also supported by the experimental inhibitory data previously reported for these inhibitors. Benzamidine showed stronger inhibitory potency against trypsin-like activity than GORE3, whereas the uncalibrated MM/GBSA and MM/PBSA estimates did not reproduce this experimental potency order.^12^ This discrepancy reinforces that the computed energetic estimates should not be used to infer absolute affinity or direct experimental potency. Instead, the molecular simulations were used here to evaluate dynamic stability, interaction persistence, and qualitative energetic behavior as part of an integrated framework that also includes enzymatic activity, larval survival, nutritional parameters, and histopathological evidence. Thus, the biological effects of GORE3 are not attributed to a stronger calculated affinity than benzamidine, but rather to the combined postingestive disruption of digestive protease homeostasis, inefficient compensatory responses, impaired nutritional performance, and midgut tissue damage.

In addition, the computational analysis was designed to provide a focused mechanistic view of representative trypsin–inhibitor interactions selected from a previously published docking study. Accordingly, the simulated systems should be interpreted as molecular models describing the dynamic behavior of selected complexes, rather than as an exhaustive representation of the complete diversity of trypsin-like isoforms present in the larval midgut. This scope is biologically relevant because lepidopteran larvae may express multiple digestive protease isoforms, including inhibitor-insensitive variants, as part of compensatory responses. Therefore, the molecular dynamics results were interpreted as complementary mechanistic evidence and integrated with enzymatic, physiological, nutritional, survival, and histopathological analyses.

The comparison between the *in silico* and *in vivo* results reveals an important feature of GORE3.^12^ Although SKTI showed the most favorable binding free-energy values and the highest number of hydrogen bonds, GORE3 produced the strongest negative effects on larval survival and larval weight.^6,9^ This indicates that the biological impact of a protease inhibitor in *S. frugiperda* cannot be predicted solely from binding free energy or the number of hydrogen bonds.^10,12^ In a digestive system characterized by multiple protease isoforms, compensatory enzyme production, and physiological plasticity, the effect of an inhibitor depends not only on binding strength but also on how its interaction perturbs digestive regulation over time.^5,42^

Regarding GORE3, molecular data suggest a stable binding interaction, whereas biochemical and physiological data indicate that this interaction is linked to digestive imbalance. The peptide elicited a significant increase in trypsin-like and total protease activity at a later point, implying a compensatory response by the larvae.^43,44^ Nonetheless, this response did not restore normal performance, as evidenced by decreased larval weight, reduced food-conversion efficiency, altered survival rates, and severe midgut damage. Consequently, GORE3 appears to induce a discrepancy between digestive compensation and physiological recovery: larvae augment proteolytic activity; however, this increase is insufficient to sustain normal growth and tissue integrity.^45,46^

Trypsin-like activity exhibited a dynamic response to the treatment: an initial decrease in enzymatic activity, followed by a subsequent increase, especially in larvae exposed to GORE3. This observation indicates that the peptide not only directly interferes with digestive protease activity but also elicits a compensatory response within the digestive system. In herbivorous insects, such a response may involve overexpression of proteases, activation of inhibitor-insensitive isoforms, or reorganization of the intestinal proteolytic profile.^47,48^ In this study, the response elicited by GORE3 was more pronounced than that observed with benzamidine or SKTI, suggesting that this peptide induced a more significant disruption of proteolytic homeostasis in the midgut of *S. frugiperda*.^49^

The increase in total proteolytic activity reinforces this interpretation. Although elevated enzymatic activity may represent a physiological attempt to compensate for the initial inhibition, it does not necessarily indicate functional recovery of digestion. On the contrary, the maintenance of high protease levels may reflect a metabolically costly response, in which the insect invests energy in producing additional enzymes to preserve protein digestion. In the case of GORE3, this compensation appeared to be insufficient to restore normal larval performance, as it was accompanied by changes in nutritional parameters, reduced larval weight, and increased mortality,^10,50,51^

The nutritional indices indicate a dissociation between apparent digestion and the efficient conversion of nutrients into biomass. The increase in approximate digestibility (AD), together with the reduction in the efficiency of conversion of ingested food (ECI), suggests that larvae exposed to GORE3 were able to process part of the ingested food but did not efficiently convert this resource into body growth. This pattern is consistent with nutritional stress induced by protease inhibitors, in which disruption of protein digestion compromises the availability or metabolic utilization of essential amino acids.^19,52^ Thus, even in the presence of an enzymatic compensatory response, the energetic cost associated with digestive reorganization may have limited mass gain and contributed to prolonged larval development.

Among the evaluated treatments, GORE3 was the only one that significantly reduced caterpillar survival, indicating a more evident biological impact than benzamidine and SKTI under the experimental conditions of this study. This result is particularly relevant because the observed physiological effect cannot be explained solely by classical inhibitory potency. Although GORE3 has higher K_i_ values than benzamidine, the molecular interaction results indicate that the peptide forms a broader contact network with trypsin-like regions and maintains stability throughout the molecular simulation.^12^ Therefore, the biological efficacy of GORE3 appears to be related not only to the strength of immediate enzymatic inhibition but also to the persistence of interactions, the disruption of proteolytic regulation, and the inability of larvae to fully compensate for digestive imbalance.^53,54^

The histopathological modifications observed in the midgut support the structural basis for the identified biochemical and physiological effects. GORE3 treatment resulted in disorganization of the midgut epithelium, including loss of cell adhesion, disruption of epithelial architecture, alterations in the muscle layer, and difficulty distinguishing cellular morphotypes. Given that the midgut is responsible for secreting digestive enzymes, absorbing nutrients, and maintaining the intestinal barrier,^55^ these alterations may directly affect the organ’s digestive function.^45^ Histopathological alterations may be used as morphological biomarkers of toxicity,^56^ including for *A. gemmatalis*.^8,57^ Despite this, corroboration of the interpretations presented here by other methods is important when the results are morphological inferences. However, qualitative histopathological data, which provide relevant clues and indicators, can be obtained in the future with semiquantitative or quantitative studies.

Despite the marked physiological and histopathological effects induced by GORE3, no significant differences were observed in cumulative leaf consumption among treatments. A similar pattern was previously reported by,^12^ who also found no reduction in foliar consumption during the first hours of exposure to GORE3. These findings suggest that the peptide does not act primarily as a feeding deterrent but rather exerts its biological effects after ingestion by disrupting digestive homeostasis.^45,58^ In this context, the absence of changes in leaf consumption, combined with reduced food-conversion efficiency, lower larval weight, delayed development, and severe midgut damage, reinforces the interpretation that GORE3 affects postingestive physiological processes rather than feeding behavior itself. This dissociation between food intake and nutritional performance is consistent with the mode of action of digestive protease inhibitors, in which insects may maintain feeding activity while experiencing impaired nutrient assimilation and elevated metabolic costs associated with compensatory digestive responses,^18,43,59^

Although GORE3 significantly reduced larval weight and prolonged larval development, pupal weight did not differ significantly from the control. At first glance, these findings may appear contradictory. However, this pattern is consistent with the developmental plasticity of holometabolous insects, in which larvae experiencing nutritional or physiological stress may delay metamorphosis until sufficient resources have been accumulated to reach the critical weight required for pupation.^60,61^ Accordingly, the prolonged larval period observed after GORE3 exposure may have provided surviving individuals with additional time to partially compensate for the initial reduction in larval biomass before pupation. Interestingly, the same pattern was observed in our previous study evaluating GORE3 against *S. frugiperda*, in which the peptide significantly reduced larval body mass and extended larval development without affecting pupal weight, despite causing substantial mortality and marked physiological impairment (Figure 7).^12^ This reproducibility across independent experiments suggests that the maintenance of pupal weight is a consistent biological response rather than an isolated observation. Importantly, because GORE3 significantly reduced survival, pupal weight was necessarily measured only in individuals that successfully completed larval development, representing the most tolerant fraction of the original population. Therefore, the absence of differences in pupal weight should not be interpreted as complete physiological recovery but rather as the combined consequence of compensatory developmental growth and survivor selection. This interpretation is supported by the concomitant reductions in survival, larval body mass, and development, and the impaired digestive efficiency and midgut histopathological alterations observed in the present study.

The oxidative stress response appears to have played a complementary, but not central, role in larval responses to the inhibitors. Changes in catalase and total antioxidant capacity were observed at specific instars; however, other biomarkers, such as hydrogen peroxide, nitric oxide, carbonylated proteins, and SOD, were more influenced by larval stage than by treatment. These results indicate that the redox state may contribute to the general physiological stress response, but it does not appear to be the primary explanatory axis for the effects of GORE3. Therefore, the data more convincingly suggest that digestive dysregulation and structural impairment of the midgut are central processes associated with decreased larval performance.

A limitation of the computational analysis is that only one representative *S. frugiperda* trypsin-like isoform was modeled, whereas lepidopteran midguts contain multiple trypsin-like proteases and may express inhibitor-insensitive isoforms during compensatory responses. Therefore, the simulated complex should be interpreted as a mechanistic model of one possible trypsin–inhibitor interaction rather than as a complete representation of the full digestive protease repertoire. In addition, the MM/GBSA and MM/PBSA calculations were performed without explicit entropic correction and were therefore treated as entropy-uncorrected effective energetic estimates. These values were used qualitatively and in combination with RMSD, RMSF, hydrogen-bond persistence, enzymatic assays, nutritional indices, survival analysis, and histopathology.

Overall, this study enhances understanding of GORE3 by demonstrating that its biological effects extend beyond inhibition of trypsin-like enzymes. The peptide forms a stable and energetically favorable complex with *S. frugiperda* trypsin, induces a compensatory proteolytic response, reduces nutritional efficiency, compromises midgut integrity, and impairs larval performance. The comparison between benzamidine and SKTI indicates that the in vivo effect of a protease inhibitor cannot be inferred solely from binding free energy or the number of hydrogen bonds; rather, it depends on how the interaction perturbs digestive regulation over time. In this context, GORE3 serves as a synthetic peptide model linking molecular recognition by digestive proteases to compensatory enzyme responses, intestinal damage, delayed development, and reduced survival in *S. frugiperda*.

## 5 Conclusion

This study shows that GORE3 disrupts the digestive physiology of *S. frugiperda* through a mechanism that extends beyond direct inhibition of trypsin-like protease activity. Molecular simulations indicated that GORE3 forms a dynamically stable trypsin complex with a consistent relative energetic profile under the applied MM/GBSA and MM/PBSA protocols. Importantly, the *in vivo* results revealed that its biological impact depends on the disruption of proteolytic regulation over time. Exposure to GORE3 induced a compensatory increase in trypsin-like and total protease activities, but this response was insufficient to preserve larval growth, nutritional efficiency, survival, and midgut integrity. The histopathological alterations observed in the midgut further indicate that digestive dysregulation is accompanied by structural damage to the tissue responsible for enzyme secretion and nutrient absorption.

Taken together, the findings endorse GORE3 as a promising synthetic peptide candidate for the development of bioinsecticides targeting *S. frugiperda*. Its efficacy appears to involve ongoing molecular interactions with digestive proteases, ineffective enzymatic compensation, diminished food-conversion efficiency, epithelial deterioration, and compromised larval fitness. Consequently, this study offers a mechanistic foundation for the rational design of peptide-based inhibitors aimed at disrupting digestive homeostasis in agricultural pests.

## Supplementary Material



## Data Availability

The data that support the findings of this study are available from the corresponding author upon reasonable request.

## Notes

Table S1, containing the physicochemical and analytical characteristics of the synthetic peptide GORE3, and Figure S1, presenting the electrospray ionization mass spectrum (ESI-MS) confirming the identity of the synthesized peptide (PDF)
