# Supplementary material for: An Autolysis-Derived Trypsin Peptide Disrupts Digestive Homeostasis and Reduces Fitness in Spodoptera Frugiperda Caterpillars
Source: ACS Omega. 2026 Aug 7;11(32):48583–601. doi: 10.1021/acsomega.6c06048 (PMC13491923; doi:10.1021/acsomega.6c06048)

Supporting Information

An Autolysis-Derived Trypsin Peptide Disrupts Digestive Homeostasis and Reduces Fitness in *Spodoptera frugiperda* caterpillars

Daniel Guimarães Silva Paulo[1,2,3], Yaremis Meriño-Cabrera[1,3], José Severiche-Castro[4], Pablo Fernandes Braga[2], Giovanna dos Santos Pereira[5], Laryssa Lemos da Silva[5], Wesley Borges Wurlitzer[6], Julia Renata Schneider[1,6], Eulálio Gutemberg Dias dos Santos[1,3], Noeli Juarez Ferla[6], José Eduardo Serrão[5], Rafael Júnior de Andrade[1,3], Otávio José Bernardes Brustolini[7]; Neilier Rodrigues da Silva Júnior[1,3], Humberto Josué Oliveira Ramos[1,3], Maria Goreti de Almeida Oliveira[1,3]*

[1] Laboratory of Enzymology and Biochemistry of Proteins and Peptides, Department of Biochemistry and Molecular Biology, Universidade Federal de Viçosa, UFV, BIOAGRO/INCT-IPP, Viçosa-MG 36570-000, Brazil

[2]Department of Entomology, Federal University of Viçosa, Minas Gerais 36570-000, Brazil

[3]Institute of Biotechnology Applied to Agriculture – BIOAGRO-UFV, Viçosa 36570-000, Brazil

[4]Departament of Física, Facultad de Ciencias Naturales y Exactas, Universidad del Valle, Cali 760032, Colombia.

[5]Department of General Biology, Universidade Federal de Viçosa, Viçosa 36570-000, Brazil

[6]Laboratory of Acarology, Tecnovates, Universidade do Vale do Taquari, Univates, Lajeado, RS 95900-000, Brazil

[7]National Laboratory for Scientific Computing, Bioinformatics Laboratory – LNCC, Petrópolis, Rio de Janeiro 25651-075, Brazil

*Corresponding Author: Prof. Dra Maria Goreti de Almeida Oliveira; malmeida@ufv.br

Address: UFV, Universidade Federal de Viçosa- Campus Viçosa, Avenida PH Rolfs

Campus Universitário, Viçosa- Minas Gerais- CEP 36570-000

Supplementary Table

Table S1. Physicochemical and analytical characteristics of the synthetic peptide GORE3.

**Property**	**Value**
Peptide code	GORE3
Amino acid sequence	LALAY
Peptide length	5 amino acids
Supplier	AminoTech (Brazil)
Molecular weight	549.6 Da
Main ion	$[M+H]^+ = 550.6$
Analytical technique	ESI-MS
Chromatography	RP-HPLC C18

Supplementary Figure

Figure S1. Electrospray ionization mass spectrum (ESI-MS) of the synthetic peptide GORE3 (LALAY-COOH). The observed protonated molecular ion at *m/z* 550.25 ($[M+H]^+$) is consistent with the expected molecular mass of approximately 549.6 Da, confirming the identity of the synthesized peptide.

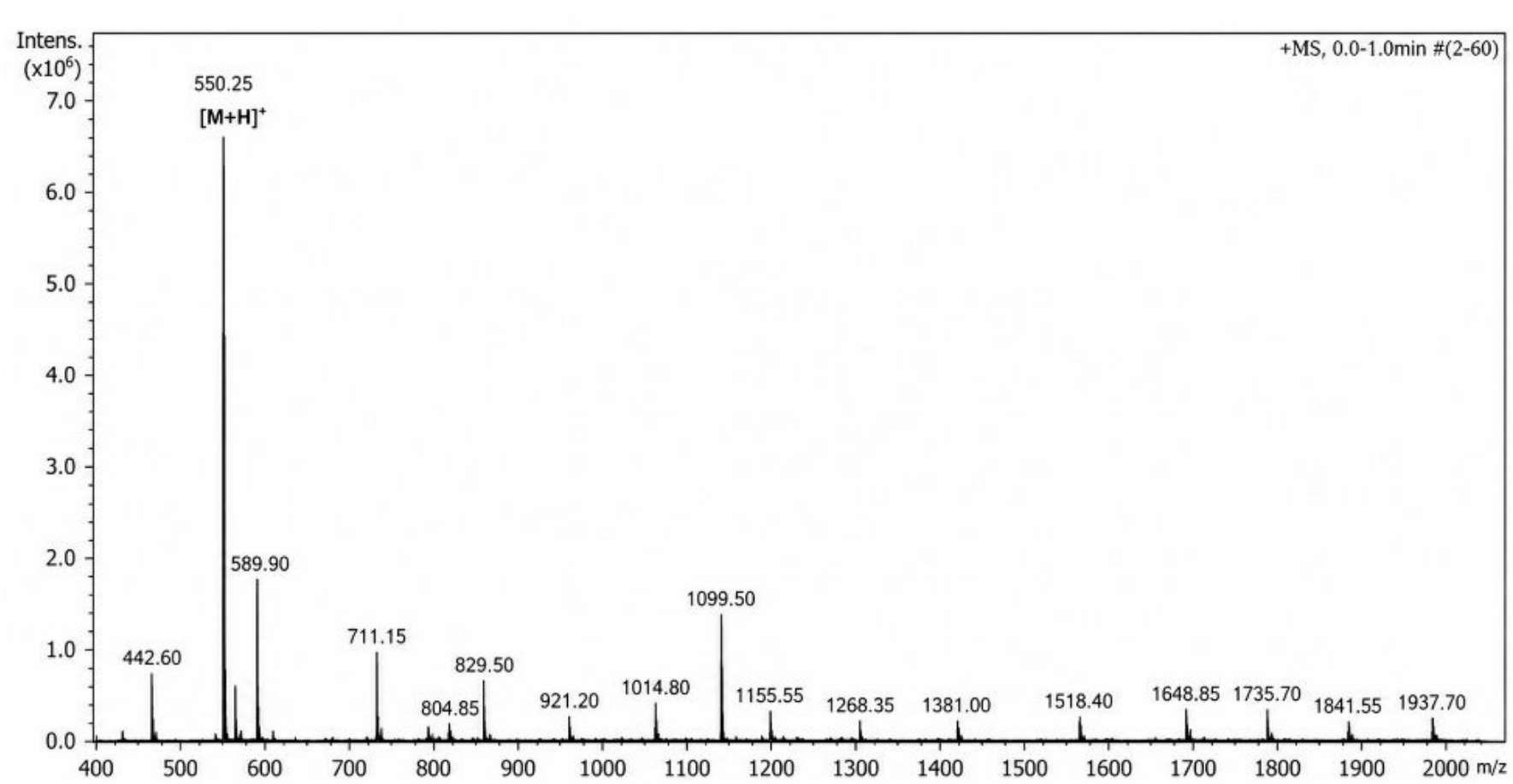

Supplement: Supplementary file 1 [file ao6c06048_si_001.pdf]
